# Development of an Algorithm to Differentiate Uterine Sarcoma from Fibroids Using MRI and LDH Levels

**DOI:** 10.3390/diagnostics13081404

**Published:** 2023-04-12

**Authors:** Ayako Suzuki, Aki Kido, Mitsuru Matsuki, Yasushi Kotani, Kosuke Murakami, Yukio Yamanishi, Isao Numoto, Hidekatsu Nakai, Tomoyuki Otani, Ikuo Konishi, Masaki Mandai, Noriomi Matsumura

**Affiliations:** 1 Department of Obstetrics and Gynecology, Faculty of Medicine, Kindai University, Osakasayama 589-8511, Japan; 2 Department of Diagnostic Imaging and Nuclear Medicine, Kyoto University, Kyoto 606-8507, Japan; 3 Department of Radiology, Faculty of Medicine, Kindai University, Osakasayama 589-8511, Japan; 4 Department of Obstetrics and Gynecology, Japanese Red Cross Wakayama Medical Center, Wakayama 640-8558, Japan; 5 Department of Gynecology and Obstetrics, Kyoto University, Kyoto 606-8507, Japan

**Keywords:** LDH, morcellator, MRI, myomectomy, uterine fibroid, uterine sarcoma

## Abstract

Background: This study aimed to establish an evaluation method for detecting uterine sarcoma with 100% sensitivity using MRI and serum LDH levels. Methods: One evaluator reviewed the MRI images and LDH values of a total of 1801 cases, including 36 cases of uterine sarcoma and 1765 cases of uterine fibroids. The reproducibility of the algorithm was also examined by four evaluators with different imaging experience and abilities, using a test set of 61 cases, including 14 cases of uterine sarcoma. Results: From the MRI images and LDH values of 1801 cases of uterine sarcoma and uterine fibroids, we found that all sarcomas were included in the group with a high T2WI and either a high T1WI, an unclear margin, or high LDH values. In addition, when cases with DWI were examined, all sarcomas had high DWI. Among the 36 sarcoma cases, the group with positive findings for T2WI, T1WI, margins, and serum LDH levels all had a poor prognosis (*p* = 0.015). The reproducibility of the algorithm was examined by four evaluators and the sensitivity of sarcoma detection ranged from 71% to 93%. Conclusion: We established an algorithm to distinguish uterine sarcoma if tumors in the myometrium with low T2WI and DWI are present.

## 1. Introduction

Uterine fibroids are a common benign disease that typically affects individuals who have reached sexual maturity. Myomectomy is the preferred surgical treatment for uterine fibroids, as it can preserve fertility. In recent years, laparoscopic myomectomy has become a popular option. A power morcellator is often used during laparoscopic myomectomy, but there is a risk that it may spread uterine sarcoma cells if this type of cancer cannot be identified before the operation [1]. While uterine sarcoma is rare, disseminating sarcoma cells in the abdominal cavity during surgery may be fatal [1]. For this reason, the US Food and Drug Administration recommends against using laparoscopic power morcellation for tumors diagnosed as uterine fibroids [1,2,3,4].

On the other hand, several reports have indicated that uterine sarcoma can be distinguished from leiomyoma through magnetic resonance imaging (MRI). A uterine sarcoma is characterized by a high T2-weighted image (WI), a diffusion-weighted image (DWI), T1WI, and unclear margins [5]. Recent studies have aimed to differentiate atypical degenerative myoma from sarcoma based on the consensus that typical myoma is easy to distinguish from sarcoma [6]. Some studies have attempted to establish a cut-off by quantifying the signals’ intensity, while others have attempted to incorporate machine learning [7,8]. However, there are complex considerations beyond merely assessing MRI, such as minimum apparent diffusion coefficient values, contrast ratio, and machine learning. Apart from the imaging findings, it is widely recognized that lactate dehydrogenase (LDH) levels are often elevated in uterine sarcoma [9].

However, most studies conducted thus far have been retrospective and have utilized small sample sizes. As a result, the procedure’s usefulness has not been thoroughly evaluated from the standpoint of excluding uterine sarcoma and safely performing surgery on uterine fibroids. Consequently, we believe that research in gynecological practice should focus on determining negative findings to establish whether the disease can be treated safely as a myoma rather than identifying positive findings of sarcoma to enhance the accuracy rate of differentiating it from degenerative myoma.

Our objective was to qualitatively evaluate basic MRI images and LDH levels for a combined total of 1801 uterine fibroids and uterine sarcomas to develop an easy-to-use algorithm capable of detecting uterine sarcomas with 100% sensitivity.

## 2. Materials and Methods

### 2.1. Study Population

This study included patients who had undergone surgery for myometrial tumors larger than 3 cm, had undergone MRI prior to treatment, and had been diagnosed pathologically with either leiomyosarcoma, low- or high-grade endometrial stromal sarcoma (ESS), or leiomyoma (excluding adenosarcoma, carcinosarcoma, and smooth muscle tumors of uncertain malignant potential).

The Kyoto cohort comprised sarcoma (*n* = 18) and leiomyoma (*n* = 1369) cases treated at Kyoto University Hospital between January 1986 and March 2005. The Kindai cohort consisted of sarcoma (*n* = 9) and leiomyoma (*n* = 293) cases treated at Kindai University Hospital between January 2011 and December 2013. For Kyoto Cohort I and Kindai Cohort I, all patients who met the criteria for leiomyoma and sarcoma were included in the study. The Kindai Cohort II comprised 10 patients who were treated at Kindai University Hospital from January 2014 to December 2018 and diagnosed with sarcoma (including one case later identified as leiomyoma, as described in the Results section) and 102 randomly selected contemporaneous leiomyoma cases.

This study was conducted with the authorization of Kindai University and Kyoto University (approval numbers R02-036 and G288). All potential study participants were allowed to decline participation by opting out.

### 2.2. MRI Protocol

Kyoto University utilized a 1.5-T MR imaging system (Signa, GE Medical Systems, Milwaukee, WI, USA, or Symphony, Siemens Healthineers, Erlangen, Germany) fitted with a phased-array coil to conduct magnetic resonance imaging scans. Standard clinical sequences comprising axial and sagittal T2WI (fast spin echo (FSE)) and sagittal T1WI (spin echo (SE)) were included. The specific parameters are presented in Table 1. Before the MRI examination, 20 mg of butyl scopolamine (Buscopan; Nippon Boehringer Ingelheim, Tokyo, Japan) was administered intramuscularly to minimize bowel motion unless contraindicated.

Kindai University used 1.5-T MR imaging systems (Signa HD xt; GE Healthcare, Milwaukee, WI, USA, or Intera Achieva, Philips Healthcare, Best, The Netherlands) fitted with phased-array coils for magnetic resonance imaging scans. Standard clinical sequences included T1-weighted SE or FSE images in the axial or sagittal planes and T2-weighted FSE images in the axial and sagittal planes. The specific parameters are listed in Table 1.

### 2.3. Review of the Literature to Determine the Evaluation Criteria for T2WI or DWI

A search was conducted on PubMed using the keywords “uterine sarcoma MRI,” which yielded 384 articles as of February 2021. Thirteen original studies that assessed uterine leiomyosarcoma or ESS by specifying comparisons with T2WI or DWI were included in the analysis.

### 2.4. Setting the Evaluation Criteria

Lesions observed by T2WI were categorized according to the Oguchi type classification, which was previously proposed [10]. If the signal intensity on T2WI was higher than that of skeletal muscle, the case was classified as having high T2WI, while a signal intensity lower than that of skeletal muscle was categorized as having low T2WI. Tumors exhibiting high T2WI signal intensity were consistent with tumors classified as Type 4 or 5 in the Oguchi type classification [10]. For T1, the signal intensity (SI) of skeletal muscles was taken as the reference standard [11]. Consequently, high SI was defined as having an SI higher than that of skeletal muscle, while low SI was defined as having a similar or lower SI than skeletal muscle. For DWI, the reference standard for SI was the normal uterine outer myometrium, which was evaluated at b = 1000 s/mm^2^. Subsequently, high SI was defined as an SI similar to or higher than that of the normal uterine outer myometrium. If the SI was high due to hemorrhage, it was assessed as high SI. Regarding the evaluation of the tumor’s border, it was deemed to be clear when the tumor margin was discernible [12]. The LDH value was determined based on whether the MRI scan and the most recent time exceeded the standard institutional value. Generally, MRI and LDH were performed one month before surgery. Our hospital used Cobas^®^ LDH IFCC Gen. 2 (Roche Diagnostics K.K.) for LDH, and the reference value was 124–222 U/L. When multiple uterine fibroids were present, a lesion larger than 3 cm with a high T2WI signal intensity was selected.

### 2.5. Method of Evaluating Inter-Examiner Agreement

The author A.S. (Reader A), an obstetrician and gynecologist with over 20 years of experience in the field, who has written a review study and possesses specialized knowledge, assessed the images of all cases in the study and formulated the diagnostic algorithm [5]. To evaluate the inter-examiner agreement for the test set, we randomly selected 31 leiomyomas with a high T2WI (including 14 algorithm-positive cases), 16 leiomyomas with a low T2WI (including 16 algorithm-positive cases), and 14 sarcoma cases with available DWI images in Kindai Cohorts I and II. A.S. apprised the other examiners of the descriptive method for T2WI, T1WI, and DWI.

Reader B was a diagnostic radiologist specializing in gynecology with over 20 years of experience. Reader C was a fellow in diagnostic radiology with less than 5 years of experience. Reader D was a gynecologist with 18 years of experience, and Reader E was an obstetrician with 15 years of experience. Readers A and D were aware of the clinical information, whereas Readers B, C, and E were not involved in the diagnosis or treatment of any of the cases in the Kindai Cohort. Readers B, C, and E were blinded to all clinical information, including the pathology results, LDH levels, and age.

### 2.6. Pathology Review

In this cohort, a case of uterine sarcoma, suspected of having been pathologically misdiagnosed, was reviewed by Dr. Sachiko Minamiguchi, a pathologist specializing in gynecological oncology at a separate institution from A.S. who was not involved in the writing of this article.

### 2.7. Statistical Analysis

Survival analysis was performed by a log-rank test. Statistical analysis was performed using Graphpad Prism version 9.0 and *p* < 0.05 was considered statistically significant.

## 3. Results

### 3.1. Determination of the Evaluation Criteria for T2WI and DWI by Reviewing Previous Reports

To evaluate the T2WI, we first reviewed previous reports and established the evaluation criteria. Most of the previous reports compared the T2WI with that of the outer myometrium when assessing tumorous lesions and we found seven articles that included 46 cases. In 2007, it was discovered that skeletal muscle has a high T2WI [13,14,15,16,17,18,19,20]. However, in one case of leiomyosarcoma reported in 2007, the T2WI was higher than that of skeletal muscle but lower than that of the myometrium. Later, in three cases of leiomyosarcoma, one had an iso-low T2WI (described as “low”) compared with the outer myometrium [20]. For endometrial stromal sarcoma (ESS), there was only one report with two patients and only one had a high T2WI compared with the outer myometrium [20]. Therefore, if the outer myometrium as is used as a criterion, detecting 100% of uterine sarcomas is impossible. On the other hand, the signal intensity of skeletal muscle was lower than that of the outer myometrium and, in all reported cases, the sarcoma had a higher signal intensity than skeletal muscle (Table 2).

Regarding DWI, when a criterion of a signal intensity greater than or equal to that of the endometrium for high DWI was used, 1 out of 4 patients with leiomyosarcoma and 2 out of 14 patients had a low DWI [14,18]. However, when the myometrium was used as the criterion, which has a lower signal intensity than the endometrium, all reported cases of sarcoma had a high signal intensity (Table 3).

For T2WI, a high signal intensity was defined as higher than that of skeletal muscle, while, for DWI, a high signal intensity was defined as higher than that of the myometrium. Specifically, DWI was defined as having a high signal intensity compared with the myometrium.

However, the margin and T1WI have not been reported to provide 100% sensitivity in diagnosing sarcoma, and their use followed previous reports (see Methods) [11,12].

### 3.2. Establishment of a Sarcoma Diagnosis Algorithm

**Figure 1:**
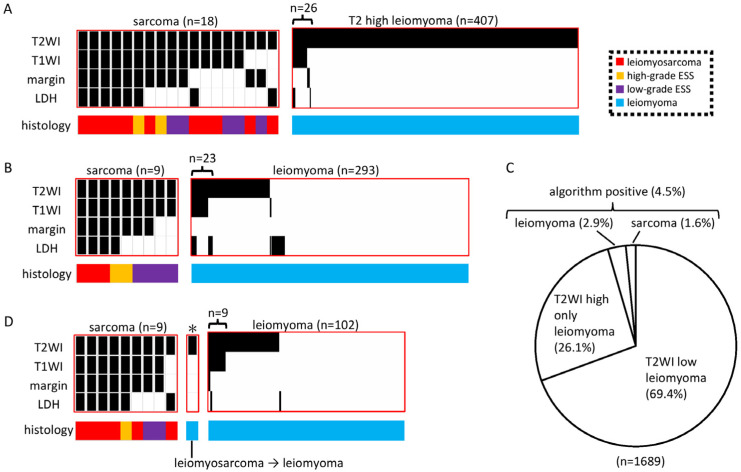
Differentiation of uterine sarcoma from uterine fibroids by MRI images and LDH levels. (**A**) Analysis of the Kyoto cohort. All 18 sarcomas had a high T2, and the others either had a high T1, unclear margins, or high LDH (the diagnostic algorithm for sarcoma). Of the 1369 myomas, 407 had a high T2. (**B**) Analysis of Kindai Cohort I. All nine sarcomas were algorithm-positive, and 23 of the 293 myomas were algorithm-positive. (**C**) Total of the Kyoto Cohort and Kindai Cohort I. Of 1689 cases, 4.5% were algorithm-positive. (**D**) Analysis of Kindai Cohort II. One case was originally diagnosed as sarcoma but was found to be myoma by a pathological review after being algorithm-negative. All other nine sarcomas were algorithm-positive and, of 102 randomly selected uterine fibroids, nine were algorithm-positive. * A case that was originally diagnosed as a sarcoma, but was found to be a myoma by pathology review after an algorithm-negative result.

A.S. (the first author of this study) subsequently developed an algorithm for diagnosing sarcoma with 100% sensitivity. Among the Kyoto cohort of 1387 cases, 18 were identified as sarcomas and all had high T2WI (as expected) compared with skeletal muscle. Furthermore, sarcomas always met the following criteria: a high T1WI, unclear margins, or high LDH. High T2WI and one of the other three factors were defined as the algorithm for detecting sarcomas with 100% sensitivity. Conversely, out of 1369 uterine fibroids, 407 had a high T2WI but only 26 were positive with the algorithm (Figure 1A).

Furthermore, Kindai Cohort I was evaluated similarly: out of 293 cases, 9 sarcoma cases were algorithm-positive, while 29 myoma cases were algorithm-positive (Figure 1B). Across Kyoto Cohort I and Kindai Cohort I, the overall percentage of algorithm-positive cases was 4.5%, with 2.9% for myoma and 1.6% for sarcoma (Figure 1C). The sensitivity of the diagnosis for sarcoma was 100%, the specificity was 97%, the positive predictive value was 36%, and the negative predictive value was 100%.

Lastly, Kindai Cohort II was also analyzed. Among the 10 cases diagnosed as sarcoma by the pathology report, 9 were algorithm-positive but 1 was algorithm-negative. Upon reviewing the pathology of the one algorithm-negative case, the diagnosis was revised to myoma. Meanwhile, out of 102 randomly selected myomas, 9 were algorithm-positive (Figure 1D).

### 3.3. Analysis of Algorithm-Positive Cases

**Figure 2:**
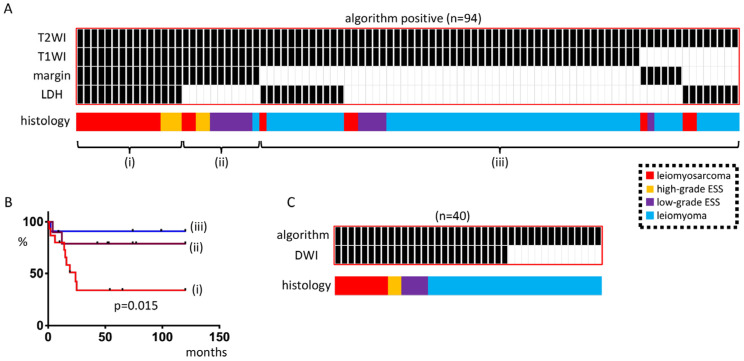
Analysis of algorithm-positive cases. (**A**) Algorithm-positive cases in the Kyoto cohort, Kindai Cohort I, and Kindai Cohort II (*n* = 94). The cases were divided into three groups: Group (i): all four factors of the algorithm were positive; Group (ii): three factors of the image were positive; Group iii: others. For Group (ii), all but one were sarcomas; for Group (iii), most were myomas, but some were sarcomas. (**B**) Prognosis differed among the three groups, with Group (i) having the poorest prognosis. (**C**) Forty of the algorithm-positive cases had available DWI and DWI was high for all sarcomas. The heatmap shows cases with a high DWI (shown in black).

We identified positive cases in all three cohorts (Kyoto Cohort I, Kindai Cohorts I and II) (*n* = 94) and observed that (i) the group with positive results for all four algorithm factors (Group 1) consisted solely of leiomyosarcoma or high-grade ESS, with no myomas present; (ii) the group with positive results for the three imaging factors of the algorithm (Group 2) consisted mostly of low-grade ESS, with only one myoma present; and (iii) the remaining group (Group 3) had more myomas (Figure 2A). Prognostic analysis revealed that Group 1, with all four factors of the algorithm being positive, had a worse prognosis compared with Group 2, with three imaging factors being positive, and the remaining group (Group 3) (*p* = 0.015) (Figure 2B). Moreover, among the algorithm-positive cases, sarcomas were found to have a high DWI in all 40 cases with available DWI images (Figure 2C).

### 3.4. Evaluation of Inter-Examiner Agreement

**Figure 3:**
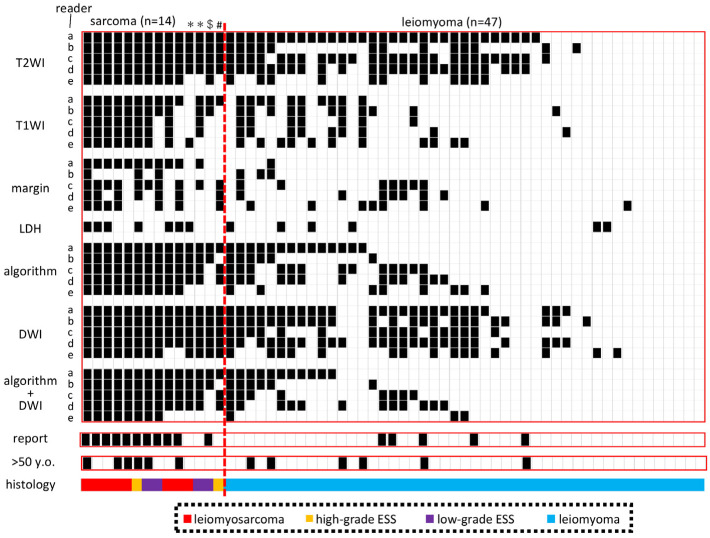
Verification of the reproducibility of image evaluation. We selected sarcoma cases with available DWI from Kindai Cohorts I and II and randomly selected algorithm-positive myomas, high-T2 myomas only, and low-T2 myomas. Algorithm + DWI was determined by taking the DWI into account. The algorithm + DWI was determined by considering the DWI and a positive algorithm but a low DWI is marked as negative in white. >50 y.o., case older than 50 years. ** The patient was followed up in the outpatient clinic for several months before surgery was performed. $ Before surgery, the LDH level was elevated, and both Readers C and D determined it to be algorithmic-positive. # The tumor protruded vaginally, and the sarcoma could be diagnosed preoperatively.

Subsequently, we evaluated whether other readers could diagnose sarcoma as algorithm-positive using the algorithm established by A.S. (Reader A) on a test set of 61 cases, including 14 sarcoma cases (Figure 3, Table 4, Supplementary Figures S1–S61 (age; pathology; final results of T2WI, T1WI, DWI, and margins as discussed by five doctors; and LHD value)). Readers B, C, and D diagnosed sarcomas as high in both T2WI and DWI with 100% accuracy, while the match rate with Reader A for T1WI and margin was only 43–86%. For Readers B, C, and D, the positive rate was 93% (13/14), which remained consistent even when low DWI was excluded. However, in the case of leiomyoma, Readers B, C, and D had lower concordance rates with Reader A than for sarcoma, ranging from 53% to 62% for T2WI and from 62% to 83% for DWI. Meanwhile, Reader E had a low algorithm-positive rate of 71% (10/14) for sarcoma but had a high concordance rate of 93% (13/14) with the original diagnostic report.

The sarcoma cases for which Readers B, C, D, and E failed to show an algorithm-positive result were initially diagnosed as sarcoma through preoperative biopsy due to a vaginally bleeding tumor. Furthermore, in the cases for which Readers C and D failed to show an algorithm-positive result, the LDH level was 217 IU/L (normal value ≤ 222 IU/L) at the time of the MRI scan but rose to 268 IU/L before the surgery, eventually showing an algorithm-positive result. Therefore, when combined with clinical information, the results of Readers B, C, and D were sufficient to suspect sarcoma preoperatively in all sarcoma cases. Reader E’s results demonstrated that, if high T2WI or high DWI were considered to be high T2WI, the preoperative increase in the LDH level would have produced an algorithm-positive result in all cases. However, in actual clinical practice, two of the three cases initially diagnosed as myoma when the reports were read were later found to be sarcoma after the mass grew over the next few months.

### 3.5. Treatment Strategies for Intramyometrial Masses Using Diagnostic Algorithms for Sarcoma

**Figure 4:**
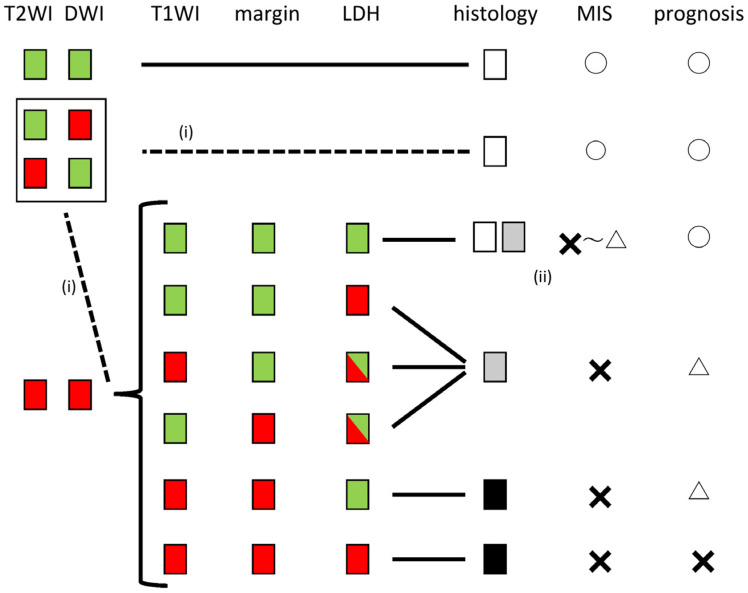
Evaluation method and treatment algorithm for intramuscular uterine masses. Red: high T2WI, high DWI, high T1WI, unclear margin, and high LDH; green indicates low values. For histology, white indicates leiomyoma, grey indicates s/o sarcoma, and black indicates sarcoma. MIS, minimally invasive surgery. (i) Normally, a low T2 or DWI indicates no sarcoma, but when evaluated by a non-gynecologic MRI specialist, either or both may be positive. However, when evaluated by a non-gynecological MRI specialist, if either of them is positive, it is safe to treat it as a case where both are positive. (ii) If both T2WI and DWI are high and the other factors are negative, the result will be negative for the algorithm. However, the reproducibility of that assessment is not 100% and does not eliminate the suspicion of sarcoma. Morcellators should be avoided or at least used within a bag.

On the basis of the data above, considering the reproducibility of the diagnosis, we outlined our recommendations for using sarcoma diagnostic algorithms for safely treating intramuscular masses (Figure 4).

Black in the heatmap indicates high T2, high T1, unclear margins, and high LDH. The type of histology is indicated by color.

## 4. Discussion

To establish a reproducible evaluation method for MRI, it is essential to make the evaluation criteria simple and clear. In many studies, the criterion for evaluating T2WI to diagnose uterine sarcoma has been whether or not the signal intensity was higher than that of the outer myometrium (Table 2). However, when the signal intensity of T2WI is compared with that of the outer myometrium, it is often low in sarcoma [20]. Furthermore, we recently found that most low-grade ESS have a low T2WI when compared with the outer myometrium (article in submission). In addition, when the criterion for DWI is the endometrium, sarcoma is often lower than that reported [16,18]. Similarly, in the present study, we found sarcomas with a low T2WI compared with the outer myometrium and a low DWI compared with the endometrium (Supplementary Figures S1–S61). Previous reports have used these criteria because many studies have tried to clarify the difference between degenerated leiomyoma and sarcoma, which have relatively high T2WI and DWI signal intensities, rather than aiming for 100% sensitivity in detecting sarcoma. The T2WI signal intensity of the myometrium varies greatly with the menstrual cycle, while that of skeletal muscle is constant and lower than that of the myometrium [8,25]. Therefore, we used skeletal muscle as the reference for T2WI and the myometrium for DWI, and we could detect sarcoma with 100% sensitivity in four patients, except for Reader E (Figure 3). Considering the risk of parasitic myoma, a power morcellator may be acceptable while using a containment system [1,2,3,4].

In the present study, Reader A had no sarcoma cases when both T2WI and DWI were high but T1WI was low, the margin was clear, and LDH was low, while Readers B, C, and D each had one sarcoma case in this group (Figure 3). These “mixed” sarcoma cases were cases that could have been diagnosed with a suspected sarcoma preoperatively due to the clinical course but it would be dangerous to assume that sarcoma was not included in this category at this time. Therefore, minimally invasive surgery (MIS) using a power morcellator should be avoided as much as possible after discussing this with the patient. If it is chosen, it should be used carefully with a containment system. If T2WI and DWI are high, with either a high T1WI, unclear margins, or a high LDH, sarcoma should be suspected as algorithm-positive (Figure 1). In such cases, guidelines and MIS should not be performed, as this would be a case of suspected malignancy [1,2,3,4]. In particular, if all three imaging findings are positive, the possibility of sarcoma is extremely high, and if the LDH is high, the prognosis may be poor (Figure 2). Consistent with our results, it has been reported that an elevated LDH expression in uterine sarcoma correlated with a poor prognosis [26]. Thus, our simple algorithm can be reproduced even by non-specialists in gynecological MRI imaging and is directly relevant to medical treatment policy (Figure 4).

This study had several limitations. Firstly, it was a retrospective study. Secondly, the sample size was relatively small for verifying the high sensitivity of detection, as there were only 36 cases of sarcoma in the cohort, while there were 1801 cases in the entire cohort. Thirdly, the data are heterogeneous, as we used images from an older era. Fourthly, smooth muscle tumors of uncertain malignant potential were excluded. Fifthly, the sensitivity of the algorithm for detecting sarcoma was not 100% in the study of inter-observer agreement. In the present study, even when limited to sarcoma, the agreement rate for evaluating individual factors was low, especially for the margins (Table 4). One of the reasons for this result was that the criteria were communicated to the narrative and evaluated on the basis of that instead of using actual MRI images in the training set. It should also be noted that, in Japan, the number of MRIs per capita is much larger than that in other countries, and almost all cases of surgery for the diagnosis of uterine fibroids or uterine sarcoma are performed preoperatively by MRI without contrast [27].

## 5. Conclusions

We have proposed the first diagnostic algorithm to identify sarcoma with close to 100% sensitivity based on our sample size. Once this algorithm has been established and commonly used, it is expected to minimize the risk of latent uterine sarcoma and allow safe treatment, including MIS.

## Figures and Tables

**Table 1:** Magnetic resonance imaging protocols.

			Kyoto 1	Kyoto 2		Kinki 1	Kinki2
		Machine	1.5 T, SIGNA, GE Healthcare	1.5 T, Symphony, Siemens		1.5 T, Signa HD xt, GE Healthcare	1.5 T, Intera Achieva, Philips Healthcare
T1WI	Sagittal	Sequence	SE	SE	Axial or Sagittal	SE or FSE	SE or FSE
		TR/TE	500/25	590/12		450–481/7–9	450–481/7–9
		Thickness	5	5		5	5
		FOV	320	320		220–360	220–360
		Matrix	512–256 × 192–256	NA		320 × 256, 512 × 512	320 × 256, 512 × 513
T2WI	Axial	Sequence	SE or FSE	TSE	Axial or Sagittal	FSE	FSE
		TR/TE (ms)	2000/40 ms	5400/120 ms		2134–8057/79–106	2134–8057/79–106
		Thickness (mm)	5	5		5	5
		FOV (cm)	320	320		220–420	220–420
		Matrix	NA	NA		384 × 384, 512 × 512	384 × 384, 512 × 512
T2WI	Sagittal	Sequence	SE or FSE	TSE			
		TR/TE (ms)	2000/40 ms	3730–7830/105–120			
		Thickness (mm)	5	5			
		FOV (cm)	320	208			
		Matrix	256 × 192–258	256 × 320, 320 × 320			
DWI		Sequence			Axial	SE/EPI	SE/EPI
		b values (s/mm^2^)				1000	1000
		TR/TE (ms)				4000–6654/54–78	4000–8654/54–78
		Thickness (mm)				5	5
		FOV (cm)				35–42	35–42
		Matrix				128 × 192–256 × 256	128 × 192–256 × 256

SE, spin echo; TSE, turbo spin echo; T, tesla; FSE, fast spin echo; EPI, echo planar imaging.

**Table 2:** The signal intensity of uterine sarcoma for T2WI.

Comparison with	Histology	High	Low	Year	Reference
Outer myometrium	LMS	9	0	2004	Tanaka et al. [13]
		0	1	2007	Lakhman et al. [20]
		6	0	2008	Tamai et al. [14]
		3	1	2010	Cornfeld et al. [12]
		4	0	2009	Namimoto et al. [15]
		6	0	2015	Tasaki et al. [16]
		6	0	2016	Lin et al. [17]
		9	10	2017	Lakhman et al. [20] (a)
		11	8	2017	Lakhman et al. [20] (b)
		16	0	2017	Li et al. [18]
		3	0	2019	Takeuchi et al. [19]
	ESS	1	1	2008	Tamai et al. [14]
		1	1	2010	Cornfeld et al. [12]
		2	0	2009	Namimoto et al. [15]
		1	0	2019	Takeuchi et al. [19]
Skeletal muscle	LMS	1	0	2007	Fukunishi et al. [21]
		3	0	2013	Thomassin-Naggara et al. [22]
	ESS	11	0	2013	Thomassin-Naggara et al. [22]

**Table 3:** The signal intensity of uterine sarcoma for DWI.

Comparison with	Histology	High	Low	Year	Reference
Endometrium	LMS	4	1	2008	Tamai et al. [14]
		5	0	2014	Sato et al. [23]
		14	2	2017	Li et al. [18]
	ESS	2	0	2008	Tamai et al. [14]
Myometrium	LMS	6	0	2015	Tasaki et al. [16]
		6	0	2016	Lin et al. [17]
		3	0	2019	Takeuchi et al. [19]
	ESS	1	0	2019	Takeuchi et al. [19]
Skeletal muscle	LMS	8	0	2015	Sumi et al. [24]
	ESS	6	0	2015	Sumi et al. [24]

**Table 4:** Percentage agreement between Readers A and B or versus the report.

	Reader	vs. Reader A			vs. Reader B			vs. Report		
		Sarcoma	Leiomyoma	Overall	Sarcoma	Leiomyoma	Overall	Sarcoma	Leiomyoma	Overall
T2WI	b	100%	53%	64%						
	c	100%	57%	67%	100%	62%	70%			
	d	100%	62%	70%	100%	66%	74%			
	e	79%	70%	72%	79%	87%	85%			
T1WI	b	86%	89%	89%						
	c	64%	79%	75%	86%	91%	90%			
	d	64%	79%	75%	86%	87%	87%			
	e	64%	79%	75%	71%	70%	70%			
Margin	b	43%	96%	84%						
	c	71%	70%	70%	50%	83%	75%			
	d	50%	66%	62%	71%	83%	80%			
	e	50%	68%	64%	57%	77%	72%			
Algorithm	a							79%	60%	64%
	b	93%	79%	82%				86%	77%	79%
	c	93%	74%	79%	86%	72%	75%	71%	74%	74%
	d	93%	74%	79%	86%	72%	75%	71%	74%	74%
	e	71%	68%	69%	79%	83%	82%	93%	81%	84%
DWI	b	100%	83%	87%						
	c	100%	66%	74%	100%	72%	79%			
	d	100%	62%	70%	100%	81%	85%			
	e	86%	66%	70%	86%	68%	72%			
DWI + algorithm	a							79%	66%	69%
	b	93%	85%	87%				86%	77%	79%
	c	93%	77%	80%	86%	83%	84%	71%	81%	79%
	d	93%	72%	77%	86%	70%	74%	71%	77%	75%
	e	57%	74%	70%	64%	85%	80%	79%	83%	82%

## Data Availability

Not applicable.

## Supplementary Materials

The following supporting information can be downloaded at https://www.mdpi.com/article/10.3390/diagnostics13081404/s1. Figures S1–S61. Cases 1–61: (A) T2WI, (B) T1WI, (C) DWI, (D) image evaluations of Readers A–E.
