# Supplementary material for: Development of an Algorithm to Differentiate Uterine Sarcoma from Fibroids Using MRI and LDH Levels
Source: Diagnostics (Basel). 2023 Apr 12;13(8):1404. doi: 10.3390/diagnostics13081404 (PMC10137865; doi:10.3390/diagnostics13081404)
Supplement: Supplementary file 1 [file diagnostics-13-01404-s001.zip › diagnostics-2206418-supplementary.pdf]

Supplementary Figure S1. Case1 A) T2WI, B) T1WI, C) DWI, D) image evaluations of Readers a–e.

A B

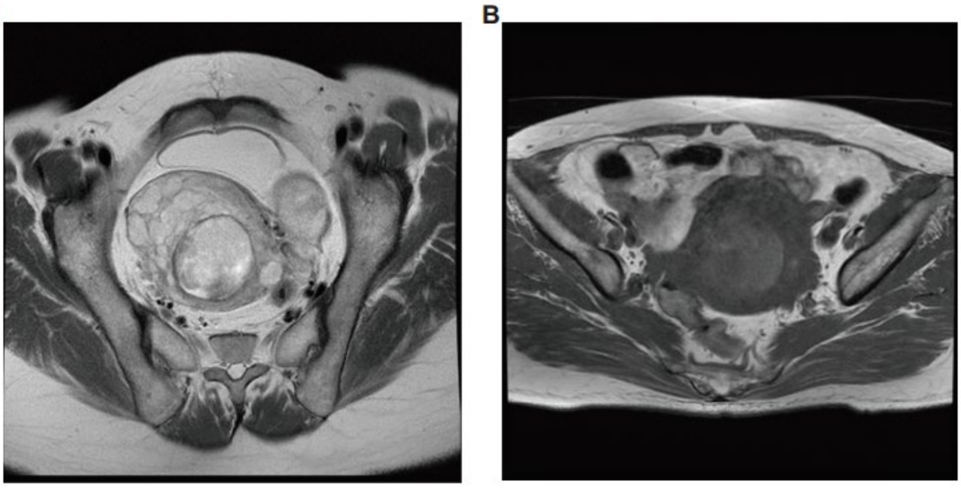

C

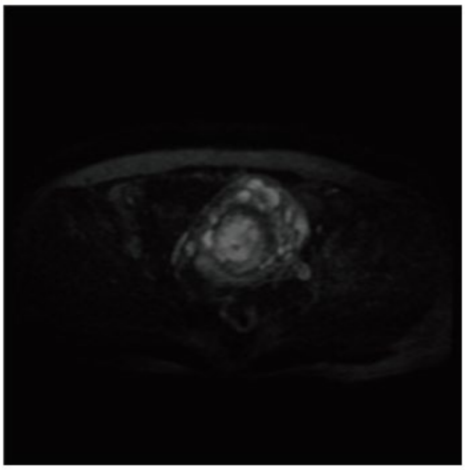

D

	T2	T1	DWI	margin
a	high	high	high	unclear
b	high	high	high	unclear
c	high	high	high	unclear
d	high	high	high	unclear
e	high	high	high	unclear

Sixty-five years old, leiomyosarcoma, high T2WI intensity, high T1WI intensity, high DWI intensity, unclear margin, LDH 226IU/L.

Supplementary Figure S2. Case2 A) T2WI, B) T1WI, C) DWI, D) image evaluations of Readers a–e.

A

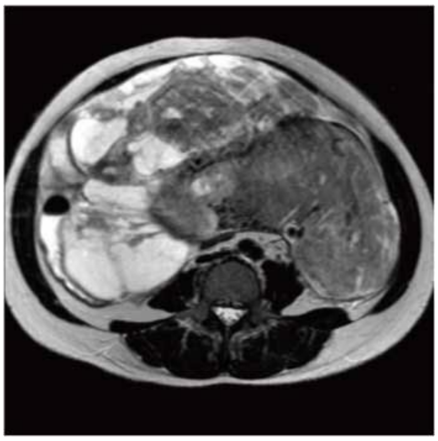

B

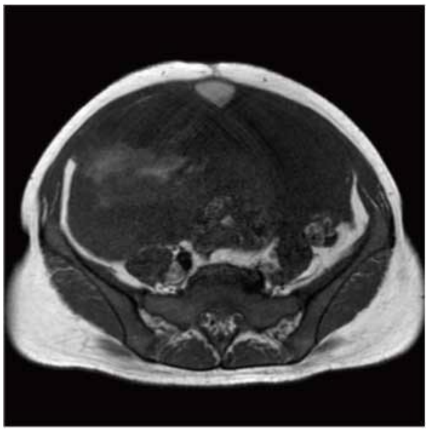

C

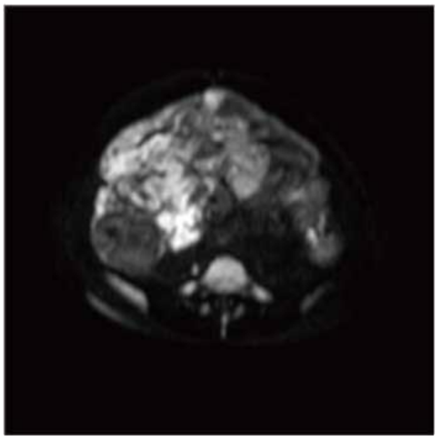

D

	T2	T1	DWI	margin
a	high	high	high	unclear
b	high	high	high	clear
c	high	high	high	unclear
d	high	high	high	unclear
e	high	high	high	unclear

Forty-four years old, leiomyosarcoma, high T2WI intensity, high T1WI intensity, high DWI intensity, unclear margin, LDH 529IU/L.

Supplementary Figure S3. Case3 A) T2WI, B) T1WI, C) DWI, D) image evaluations of Readers a–e.

A

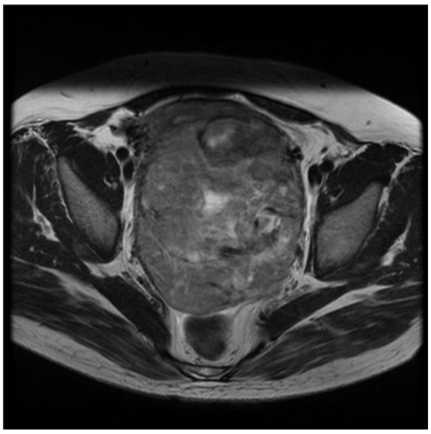

B

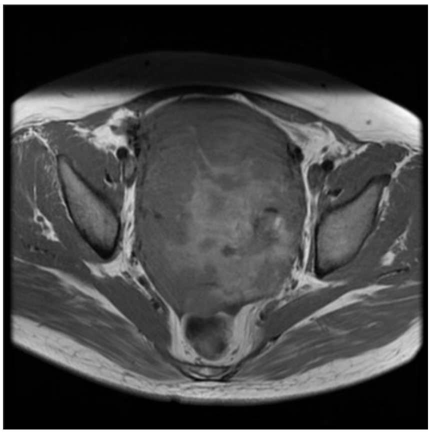

C

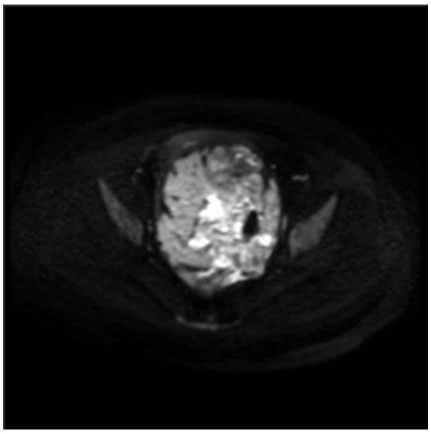

D

	T2	T1	DWI	margin
a	high	high	high	unclear
b	high	high	high	clear
c	high	high	high	unclear
d	high	high	high	clear
e	high	high	high	unclear

Forty-four years old, leiomyosarcoma, high T2WI intensity, high T1WI intensity, high DWI intensity, unclear margin, LDH 226IU/L.

Supplementary Figure S4. Case4 A) T2WI, B) T1WI, C) DWI, D) image evaluations of Readers a–e.

A

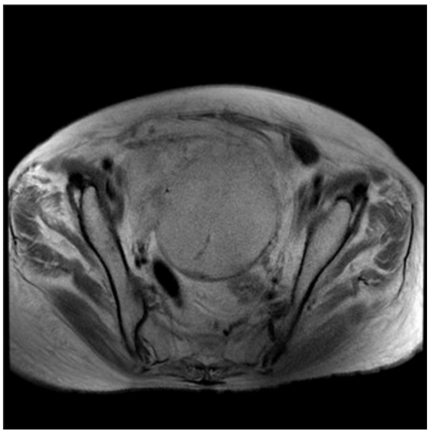

B

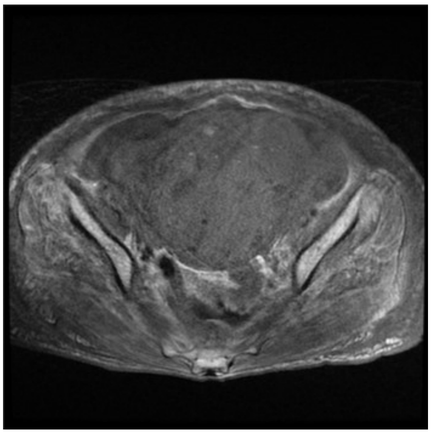

C

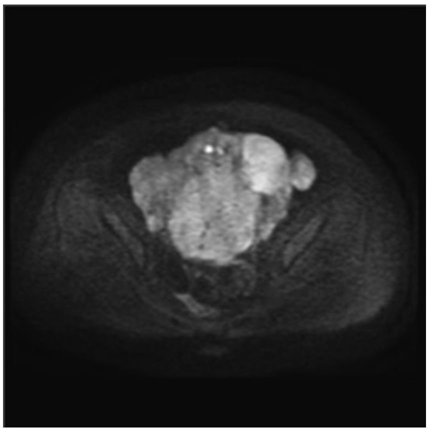

D

	T2	T1	DWI	margin
a	high	high	high	unclear
b	high	high	high	clear
c	high	high	high	unclear
d	high	high	high	unclear
e	high	high	high	unclear

Seventy-four years old, leiomyosarcoma, high T2WI intensity, high T1WI intensity, high DWI intensity, unclear margin, LDH 203IU/L.

Supplementary Figure S5. Case5 A) T2WI, B) T1WI, C) DWI, D) image evaluations of Readers a–e.

A B

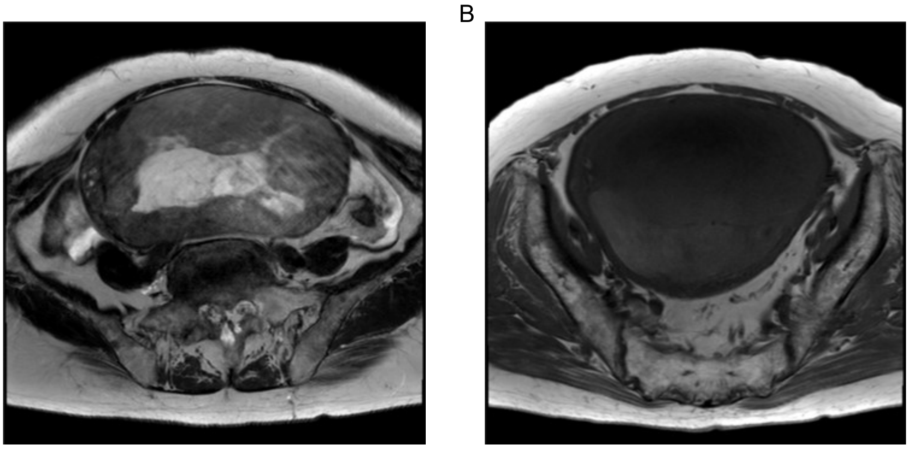

C

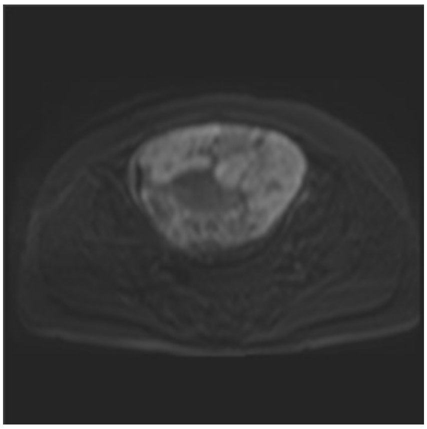

D

	T2	T1	DWI	margin
a	high	high	high	unclear
b	high	high	high	clear
c	high	high	high	clear
d	high	high	high	clear
e	high	high	high	clear

Seventy-nine years old, leiomyosarcoma, high T2WI intensity, high T1WI intensity, high DWI intensity, clear margin, LDH 222IU/L.

Supplementary Figure S6. Case6 A) T2WI, B) T1WI, C) DWI, D) image evaluations of Readers a–e.

A

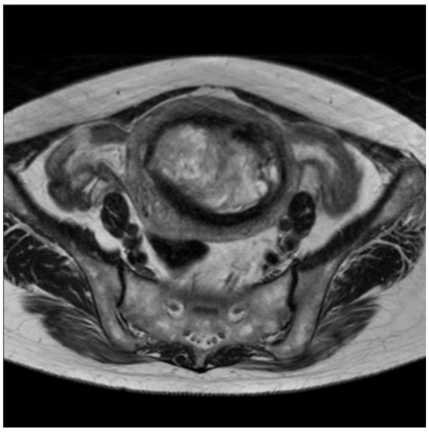

B

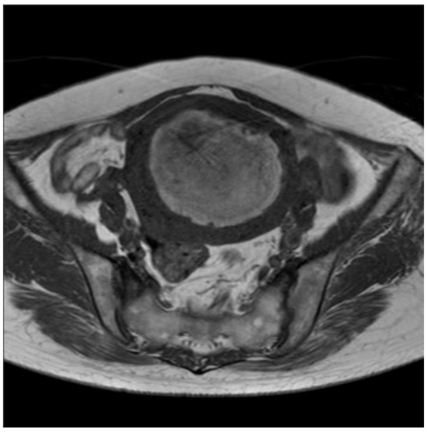

C

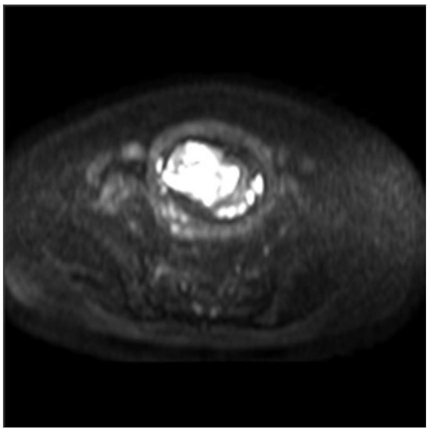

D

	T2	T1	DWI	margin
a	high	high	high	unclear
b	high	high	high	unclear
c	high	high	high	unclear
d	high	high	high	unclear
e	high	high	high	clear

Sixty-one years old, high grade ESS, high T2WI intensity, high T1WI intensity, high DWI intensity, unclear margin, LDH 462IU/L.

Supplementary Figure S7. Case7 A) T2WI, B) T1WI, C) DWI, D) image evaluations of Readers a–e.

A

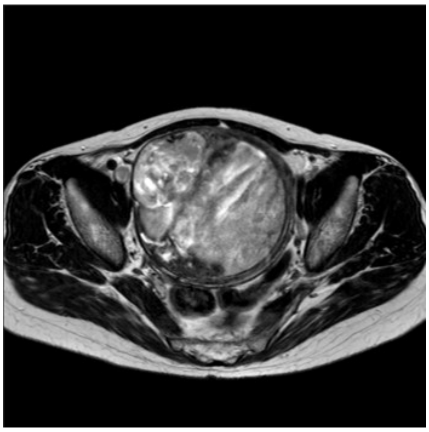

B

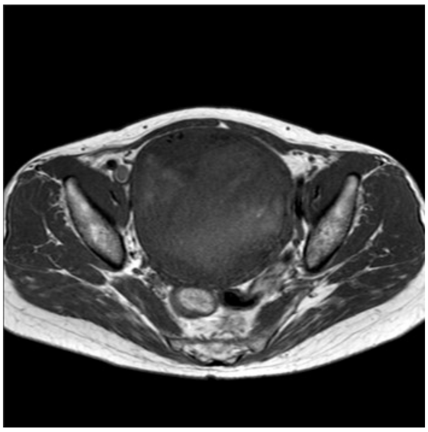

C

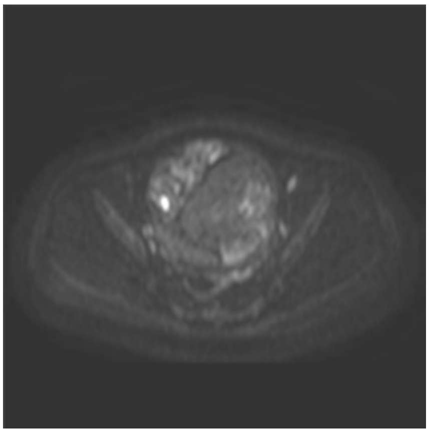

D

	T2	T1	DWI	margin
a	high	high	high	unclear
b	high	high	high	clear
c	high	high	high	unclear
d	high	high	high	clear
e	high	high	high	clear

Sixty-five years old, low-grade ESS, high T2WI intensity, high T1WI intensity, high DWI intensity, clear margin, LDH 197IU/L.

Supplementary Figure S8. Case8 A) T2WI, B) T1WI, C) DWI, D) image evaluations of Readers a–e.

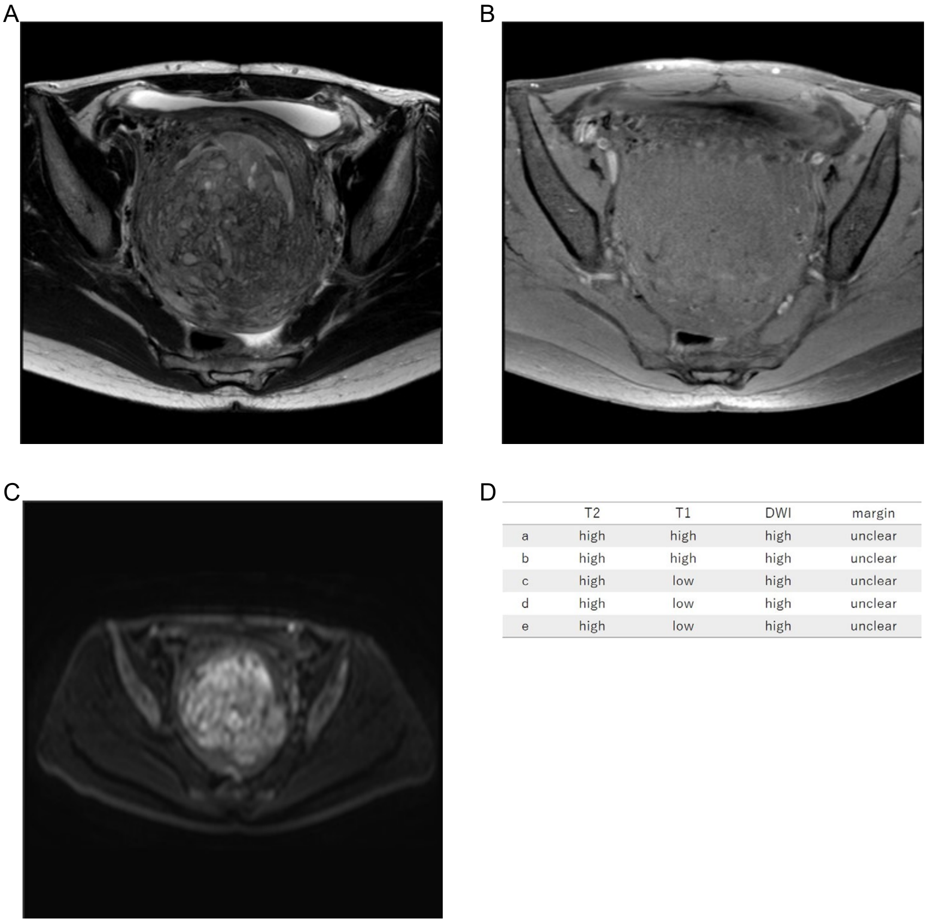

D

	T2	T1	DWI	margin
a	high	high	high	unclear
b	high	high	high	unclear
c	high	low	high	unclear
d	high	low	high	unclear
e	high	low	high	unclear

Forty-one years old, low-grade ESS, high T2WI intensity, low T1WI intensity, high DWI intensity, unclear margin, LDH 157IU/L.

Supplementary Figure S9. Case9 A) T2WI, B) T1WI, C) DWI, D) image evaluations of Readers a–e.

A

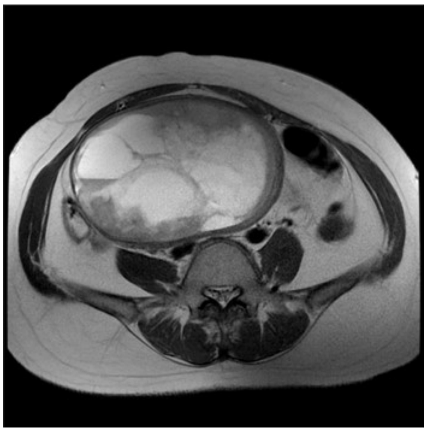

B

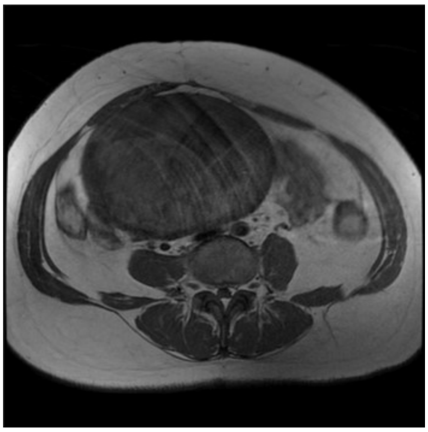

C

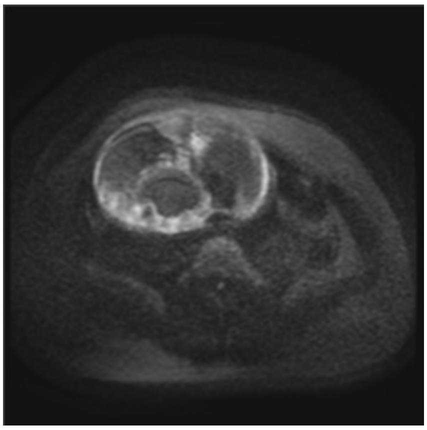

D

	T2	T1	DWI	margin
a	high	high	high	unclear
b	high	high	high	clear
c	high	low	high	clear
d	high	low	high	clear
e	high	low	high	clear

Forty-nine years old, leiomyosarcoma, high T2WI intensity, high T1WI intensity, high DWI intensity, clear margin, LDH 279IU/L.

Supplementary Figure S10. Case10 A) T2WI, B) T1WI, C) DWI, D) image evaluations of Readers a–e.

A

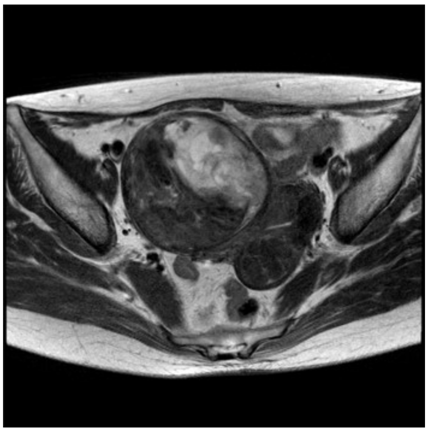

B

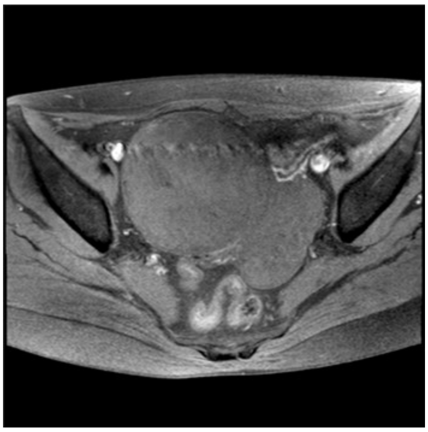

C

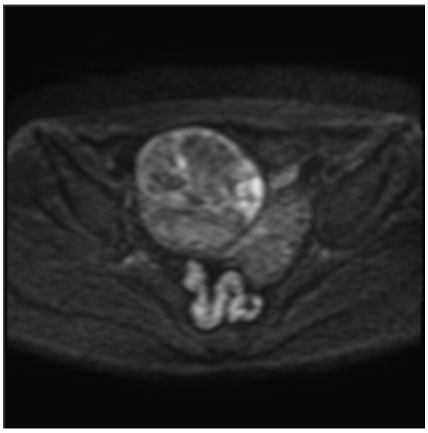

D

	T2	T1	DWI	margin
a	high	high	high	unclear
b	high	low	high	clear
c	high	low	high	unclear
d	high	low	high	unclear
e	high	low	low	unclear

Fifty-nine years old, leiomyosarcoma, high T2WI intensity, low T1WI intensity, high DWI intensity, unclear margin, LDH 228IU/L.

Supplementary Figure S11. Case11 A) T2WI, B) T1WI, C) DWI, D) image evaluations of Readers a–e.

A

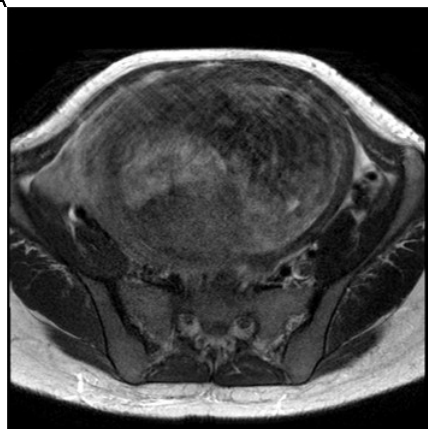

B

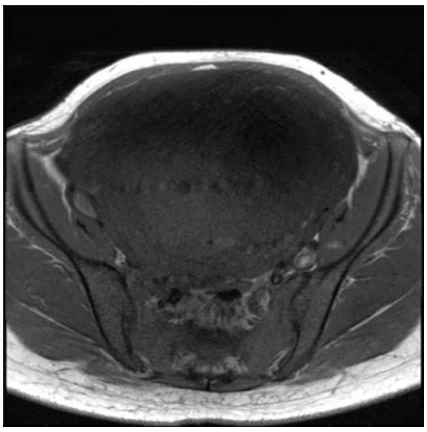

C

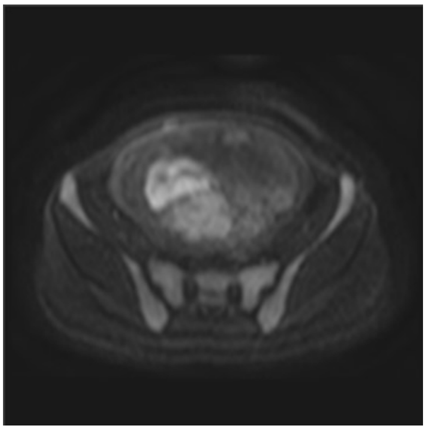

D

	T2	T1	DWI	margin
a	high	low	high	clear
b	high	low	high	clear
c	high	low	high	clear
d	high	low	high	clear
e	low	high	high	clear

Thirty-nine years old, leiomyosarcoma, high T2WI intensity, low T1WI intensity, high DWI intensity, clear margin, LDH 420IU/L.

Supplementary Figure S12. Case12 A) T2WI, B) T1WI, C) DWI, D) image evaluations of Readers a–e.

A

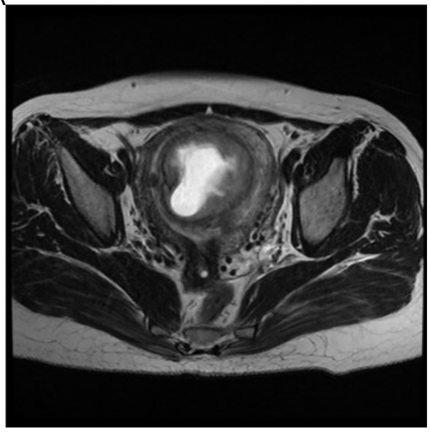

B

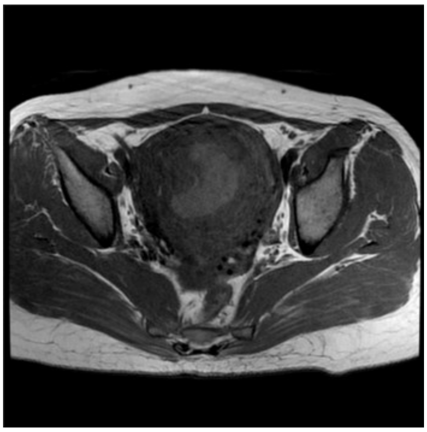

C

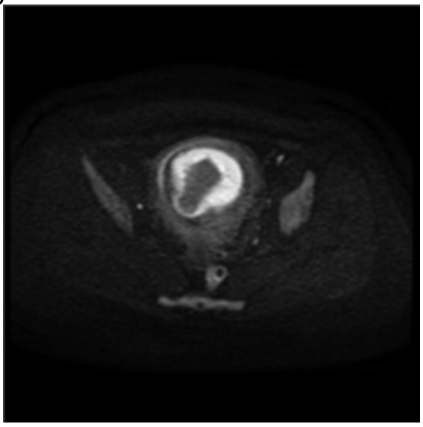

D

	T2	T1	DWI	margin
a	high	high	high	unclear
b	high	high	high	clear
c	high	high	high	unclear
d	high	high	high	clear
e	low	low	high	clear

Forty-two years old, low-grade ESS, high T2WI intensity, high T1WI intensity, high DWI intensity, clear margin, LDH 141IU/L.

Supplementary Figure S13. Case13 A) T2WI, B) T1WI, C) DWI, D) image evaluations of Readers a–e.

A

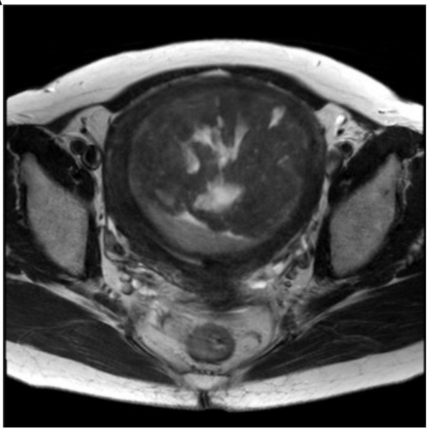

B

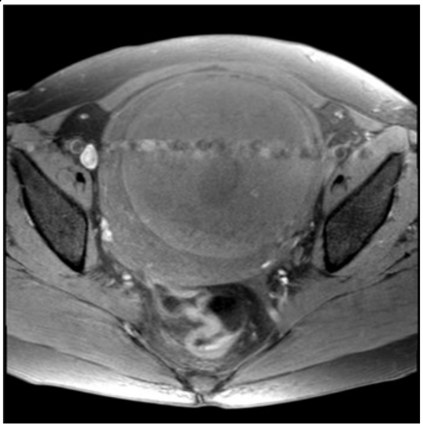

C

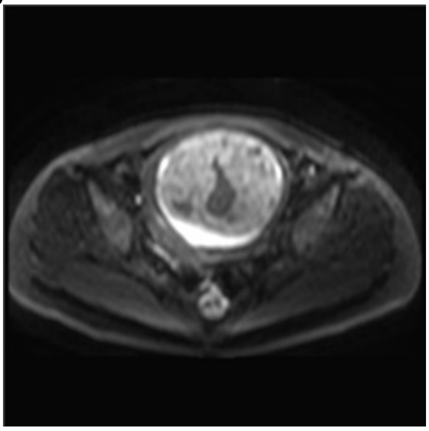

D

	T2	T1	DWI	margin
a	high	high	high	clear
b	high	high	high	clear
c	high	low	high	clear
d	high	low	high	clear
e	high	low	high	clear

Forty-four years old, low-grade ESS, high T2WI intensity, high T1WI intensity, high DWI intensity, clear margin, LDH 217IU/L.

Supplementary Figure S14. Case14 A) T2WI, B) T1WI, C) DWI, D) image evaluations of Readers a–e.

A

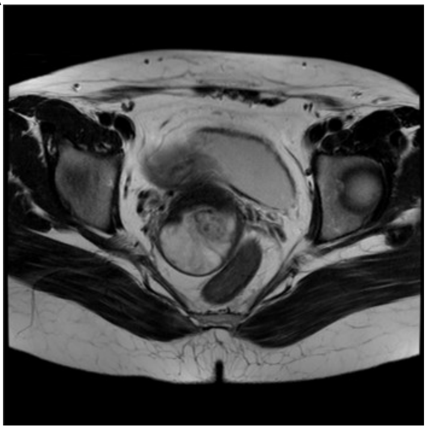

B

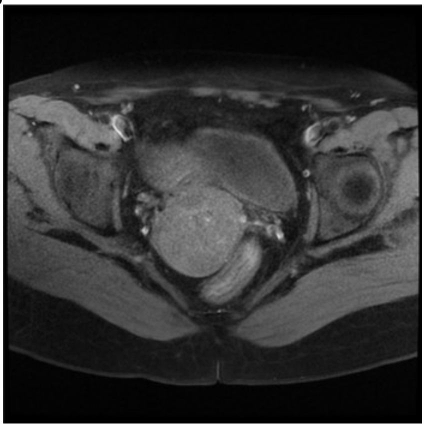

C

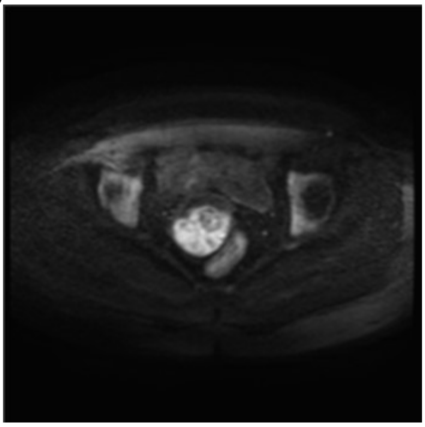

D

	T2	T1	DWI	margin
a	high	high	high	clear
b	high	low	high	clear
c	high	low	high	unclear
d	high	low	high	unclear
e	low	low	high	unclear

Forty-two years old, high-grade ESS, high T2WI intensity, low T1WI intensity, high DWI intensity, clear margin, LDH 211IU/L.

Supplementary Figure S15. Case15 A) T2WI, B) T1WI, C) DWI, D) image evaluations of Readers a–e.

A

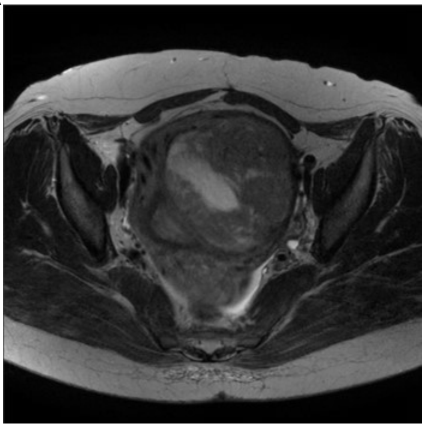

B

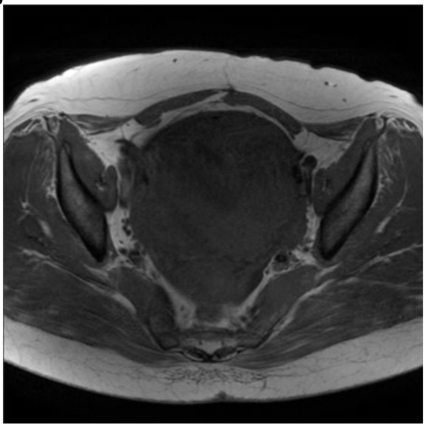

C

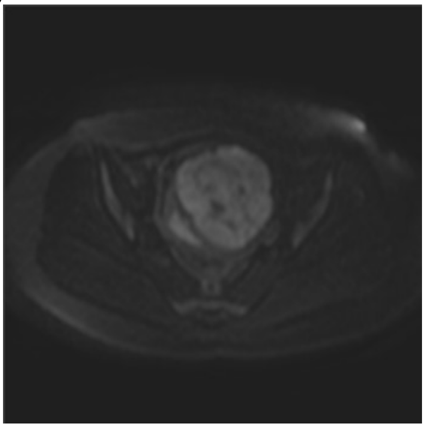

D

	T2	T1	DWI	margin
a	high	low	high	clear
b	high	low	high	clear
c	high	low	high	clear
d	high	low	high	clear
e	high	low	high	clear

Forty-two years old, leiomyoma, high T2WI intensity, low T1WI intensity, high DWI intensity, clear margin, LDH 272IU/L.

Supplementary Figure S16. Case16 A) T2WI, B) T1WI, C) DWI, D) image evaluations of Readers a–e.

A

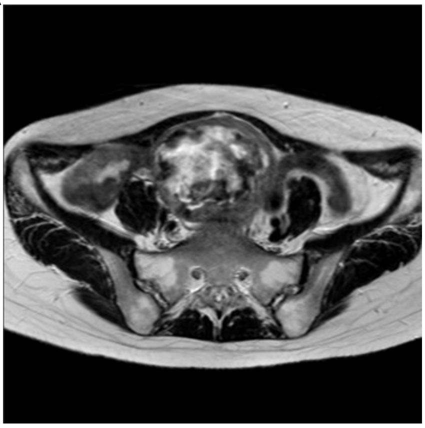

B

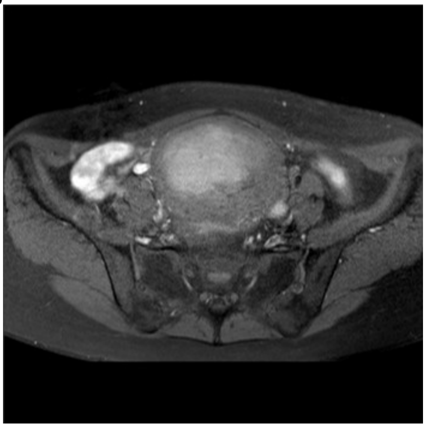

C

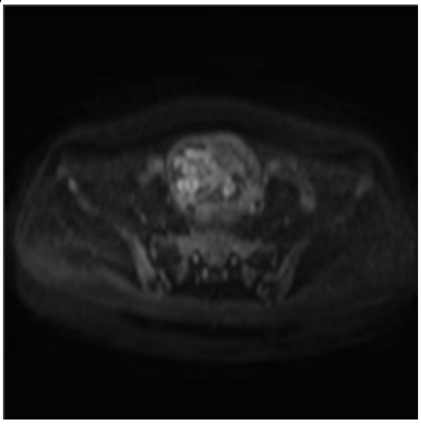

D

	T2	T1	DWI	margin
a	high	high	high	clear
b	high	high	high	unclear
c	high	high	high	unclear
d	high	high	high	unclear
e	low	high	low	clear

Thirty-one years old, leiomyoma, high T2WI intensity, high T1WI intensity, high DWI intensity, unclear margin, LDH 158IU/L.

Supplementary Figure S17. Case17 A) T2WI, B) T1WI, C) DWI, D) image evaluations of Readers a–e.

A B

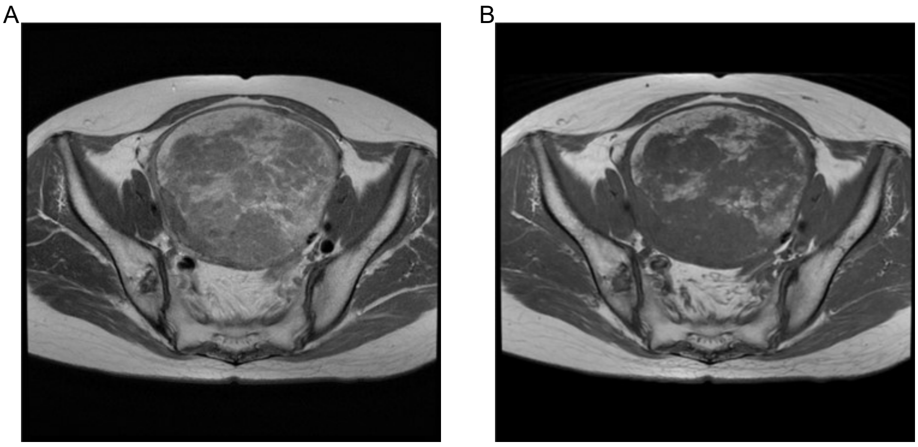

C

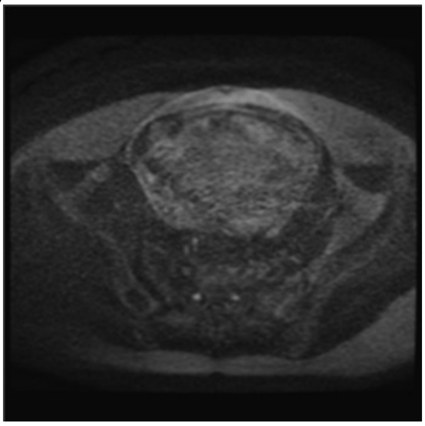

D

	T2	T1	DWI	margin
a	high	high	high	clear
b	high	high	high	clear
c	high	high	high	clear
d	high	high	low	clear
e	low	low	low	unclear

Sixty-five years old, leiomyoma, high T2WI intensity, high T1WI intensity, high DWI intensity, clear margin, LDH 194IU/L. There is some lipomyoma component within the myoma, and the T2WI is high intensity if fat is included and low intensity if fat is excluded. We finally determined the T2WI to be high intensity.

Supplementary Figure S18. Case18 A) T2WI, B) T1WI, C) DWI, D) image evaluations of Readers a–e.

A

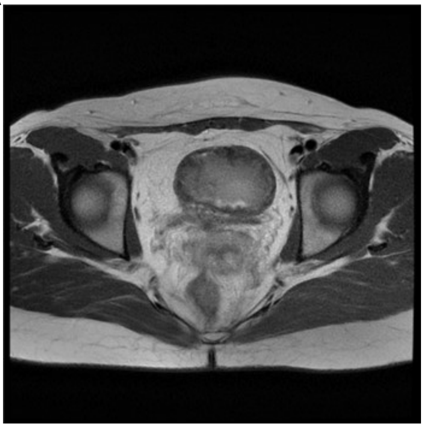

B

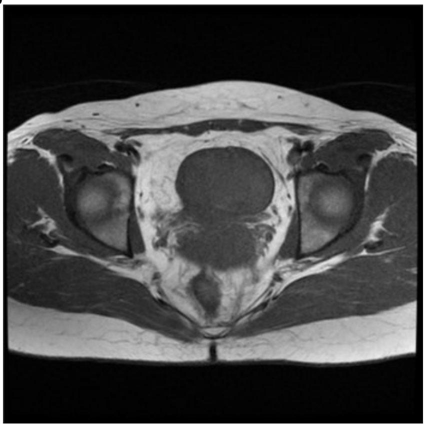

C

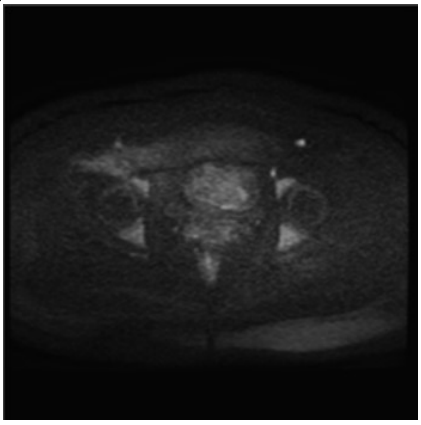

D

	T2	T1	DWI	margin
a	high	high	high	clear
b	high	low	high	unclear
c	high	low	high	clear
d	high	low	high	clear
e	high	high	low	clear

Thirty-one years old, leiomyoma, high T2WI intensity, high T1WI intensity, high DWI intensity, margin clear, LDH 170IU/L. We determined that T1WI was higher than skeletal muscle.

Supplementary Figure S19. Case19 A) T2WI, B) T1WI, C) DWI, D) image evaluations of Readers a–e.

A

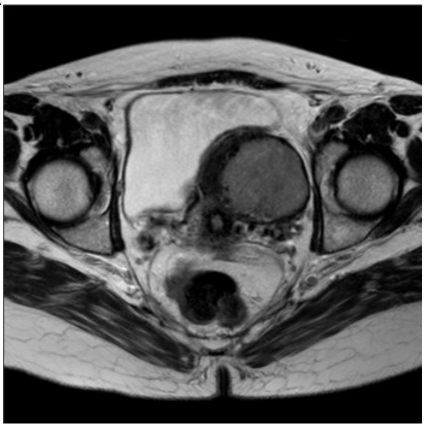

B

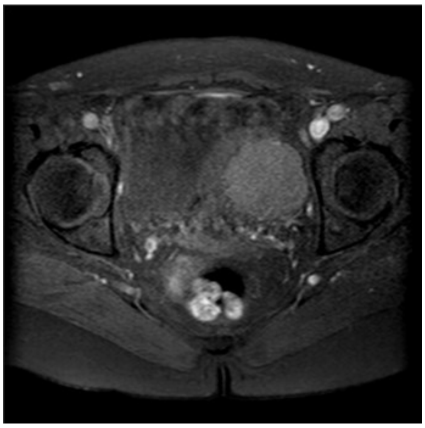

C

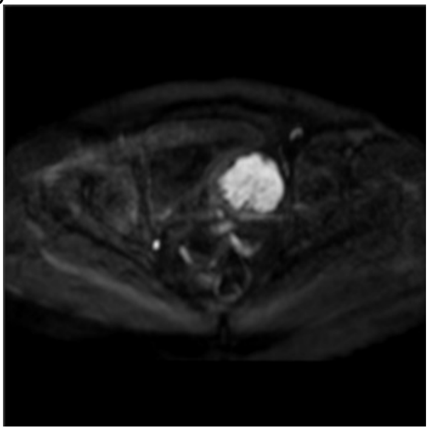

D

	T2	T1	DWI	margin
a	high	high	high	unclear
b	high	high	high	unclear
c	low	low	low	clear
d	high	low	high	clear
e	high	low	high	clear

Fifty-one years old, leiomyoma, high T2WI intensity, high T1WI intensity, high DWI intensity, clear margin, LDH 193IU/L. We reviewed the images and determined that the T2WI was Oguchi 4.

Supplementary Figure S20. Case20 A) T2WI, B) T1WI, C) DWI, D) image evaluations of Readers a–e.

A B

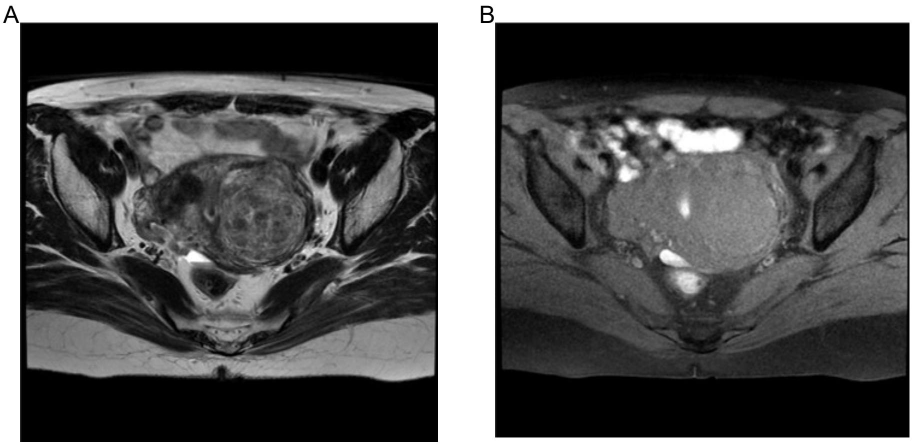

C

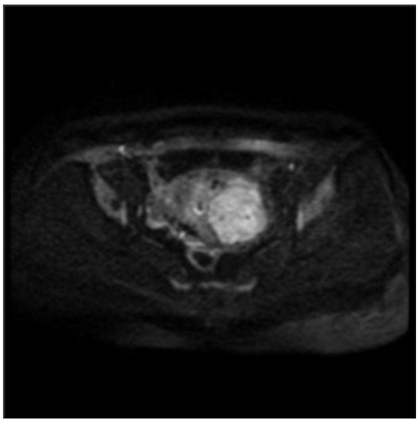

D

	T2	T1	DWI	margin
a	high	low	high	clear
b	low	low	high	clear
c	high	low	high	unclear
d	high	low	high	clear
e	low	high	high	clear

Thirty-eight years old, leiomyoma, low T2WI intensity, low T1WI intensity, high DWI intensity, clear margin, LDH 223IU/L. We reviewed the images and determined that the T2WI was Oguchi 3.

Supplementary Figure S21. Case21 A) T2WI, B) T1WI, C) DWI, D) image evaluations of Readers a–e.

A

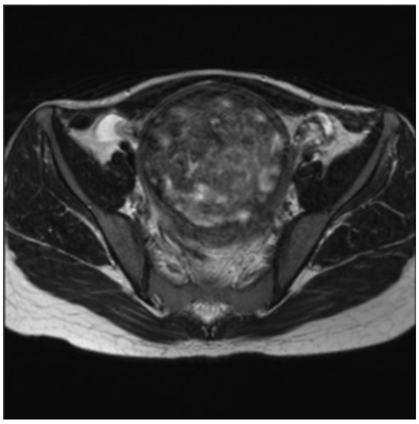

B

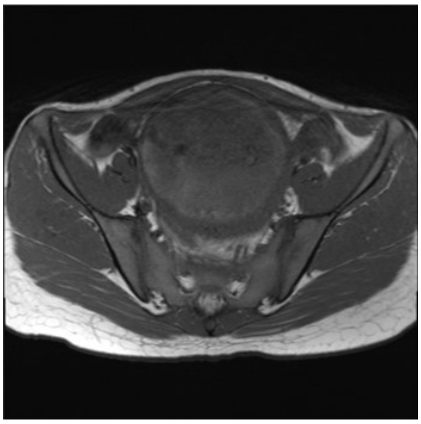

C

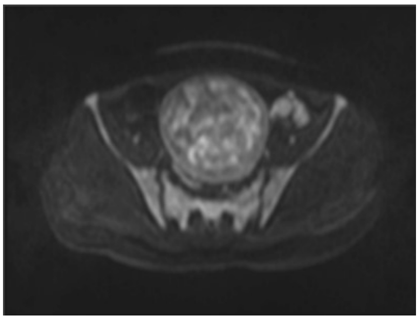

D

	T2	T1	DWI	margin
a	high	high	high	clear
b	low	high	high	clear
c	high	high	low	clear
d	high	high	high	clear
e	low	high	high	clear

Thirty-one years old, leiomyoma, low T2WI intensity, high T1WI intensity, high DWI intensity, clear margin, LDH 166IU/L. We reviewed the images and determined that the T2WI was Oguchi 3.

Supplementary Figure S22. Case22 A) T2WI, B) T1WI, C) DWI, D) image evaluations of Readers a–e.

A

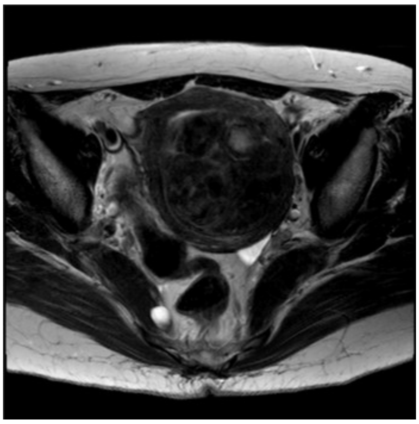

B

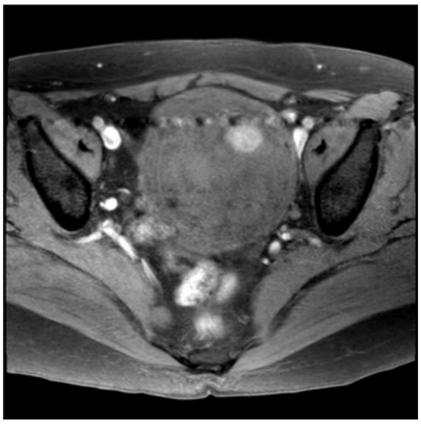

C

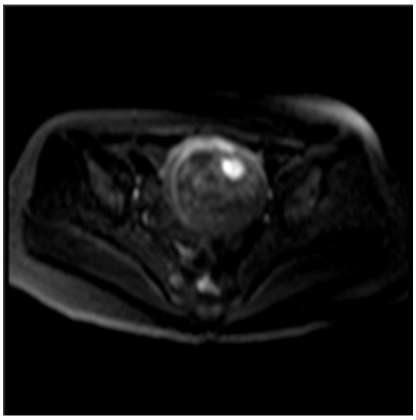

D

	T2	T1	DWI	margin
a	high	high	high	clear
b	low	high	high	clear
c	high	high	low	clear
d	high	high	high	clear
e	low	low	low	clear

Forty-two years old, leiomyoma, low T2WI intensity, high T1WI intensity, high DWI intensity, clear margin, LDH 176IU/L. We reviewed the images and determined that the T2WI was Oguchi 3.

Supplementary Figure S23. Case23 A) T2WI, B) T1WI, C) DWI, D) image evaluations of Readers a–e.

A

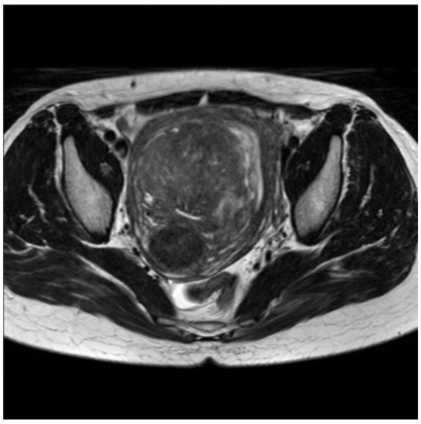

B

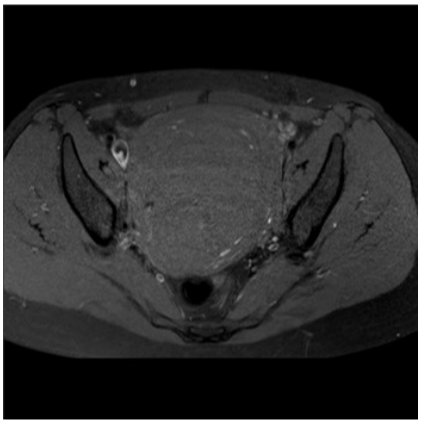

C

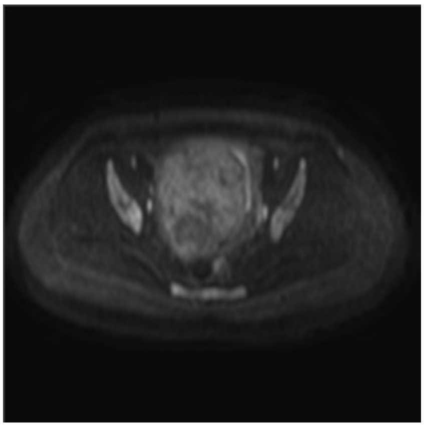

D

	T2	T1	DWI	margin
a	high	low	high	clear
b	low	low	high	clear
c	low	low	low	clear
d	low	low	low	clear
e	low	high	low	unclear

Forty-two years old, leiomyoma, low T2WI intensity, low T1WI intensity, low DWI intensity, clear margin, LDH 229IU/L. We determined that T1WI was higher than skeletal muscle.

Supplementary Figure S24. Case24 A) T2WI, B) T1WI, C) DWI, D) image evaluations of Readers a–e.

A

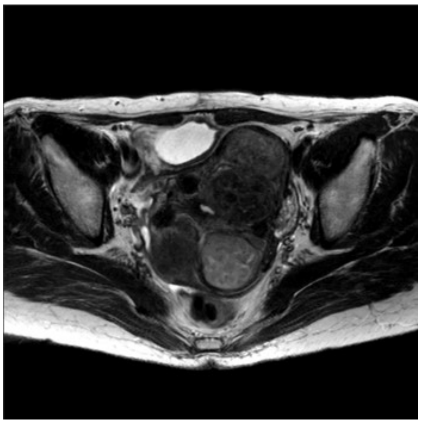

B

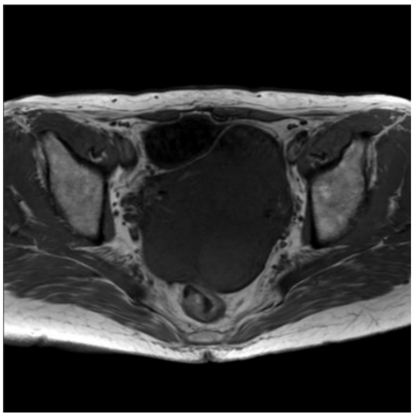

C

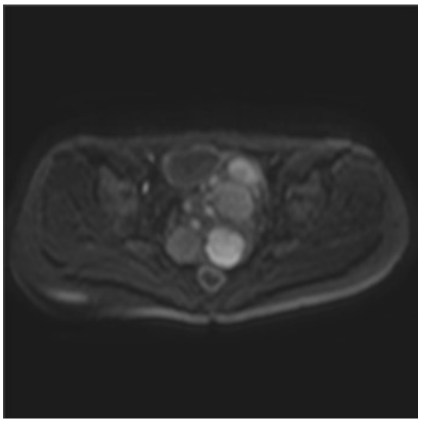

D

	T2	T1	DWI	margin
a	high	high	high	clear
b	low	low	high	clear
c	high	low	high	clear
d	high	low	high	clear
e	high	low	high	clear

Forty-eight years old, leiomyoma, high T2WI intensity, low T1WI intensity, high DWI intensity, clear margin, LDH 207IU/L.

Supplementary Figure S25. Case25 A) T2WI, B) T1WI, C) DWI, D) image evaluations of Readers a–e.

A

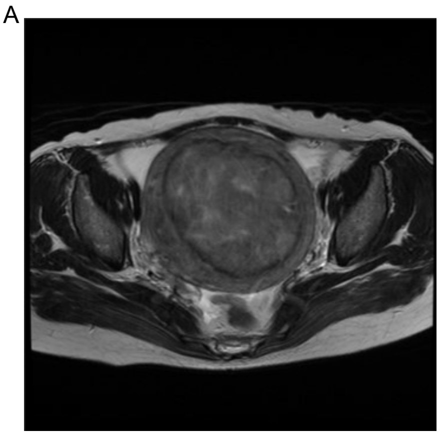

B

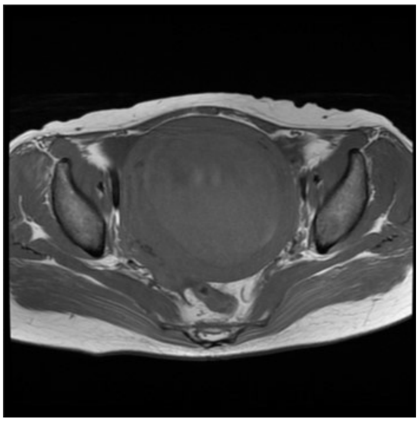

C

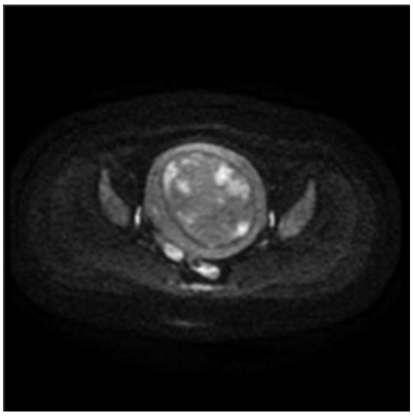

D

	T2	T1	DWI	margin
a	high	high	high	clear
b	low	high	high	clear
c	high	low	low	clear
d	low	high	high	clear
e	low	high	low	clear

Thirty-nine years old, leiomyoma, low T2WI intensity, high T1WI intensity, high DWI intensity, clear margin, LDH 170IU/L. We reviewed the images, and determined that the T2WI was Oguchi 3 and that T1WI was higher than skeletal muscle.

Supplementary Figure S26. Case26 A) T2WI, B) T1WI, C) DWI, D) image evaluations of Readers a–e.

A

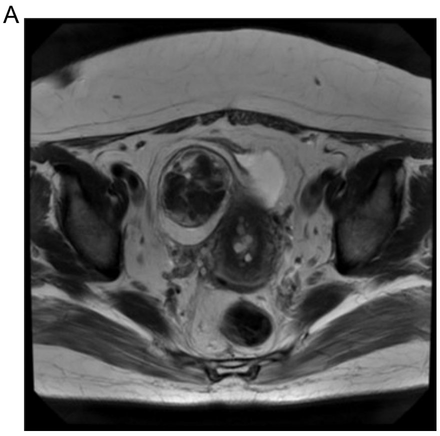

B

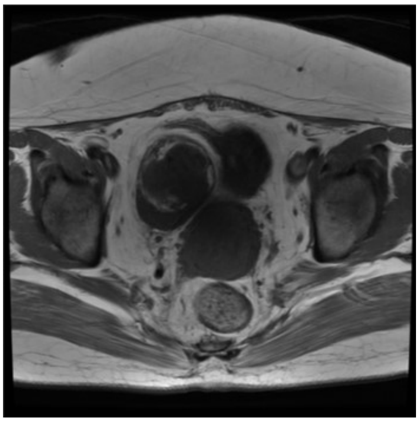

C

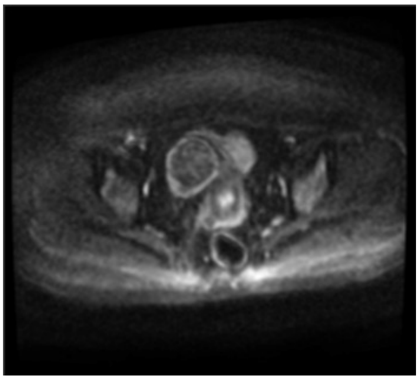

D

	T2	T1	DWI	margin
a	high	high	low	clear
b	low	high	low	clear
c	high	high	low	clear
d	high	high	high	unclear
e	low	low	low	clear

Fifty-seven years old, leiomyoma, high T2WI intensity, high T1WI intensity, low DWI intensity, clear margin, LDH 209IU/L. There is some lipomyoma component within the myoma, and the T2WI is high intensity if fat is included and low intensity if fat is excluded. We finally determined the T2WI to be high intensity.

Supplementary Figure S27. Case27 A) T2WI, B) T1WI, C) DWI, D) image evaluations of Readers a–e.

A B

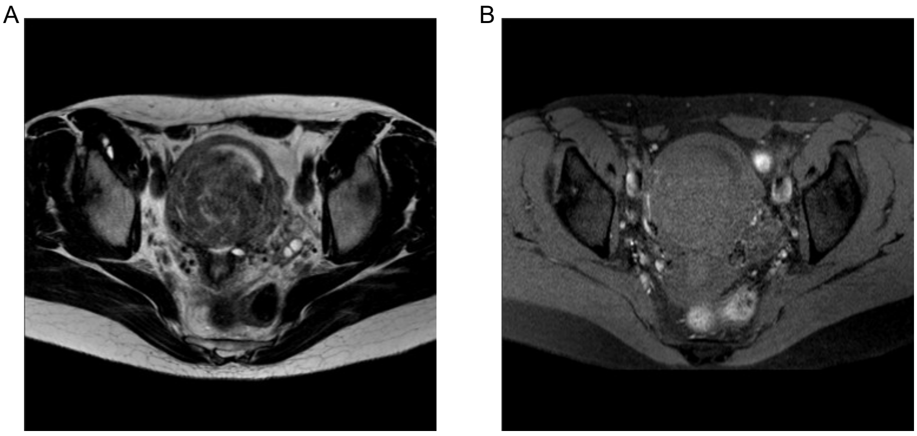

C

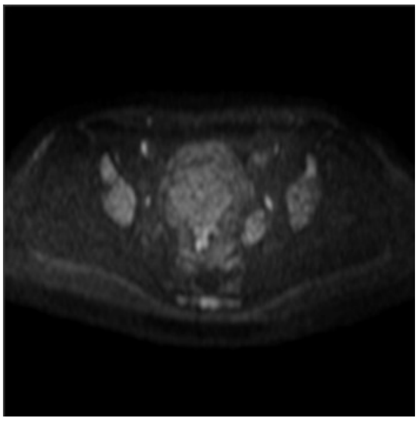

D

	T2	T1	DWI	margin
a	high	low	low	clear
b	low	low	low	clear
c	high	low	low	clear
d	low	low	low	clear
e	low	low	low	clear

Thirty-eight years old, leiomyoma, low T2WI intensity, low T1WI intensity, low DWI intensity, clear margin, LDH 223IU/L. We reviewed the images and determined that the T2WI was Oguchi 3.

Supplementary Figure S28. Case28 A) T2WI, B) T1WI, C) DWI, D) image evaluations of Readers a–e.

A

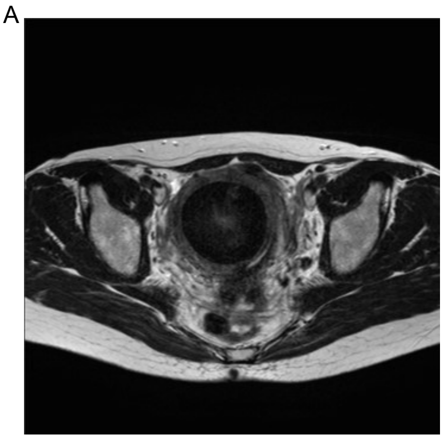

B

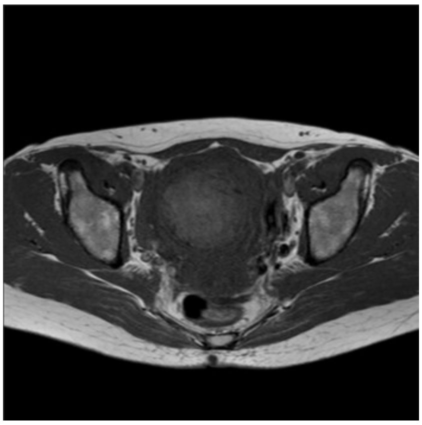

C

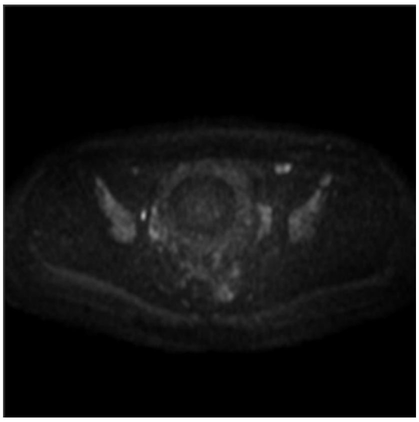

D

	T2	T1	DWI	margin
a	high	high	low	clear
b	low	high	low	clear
c	low	high	low	clear
d	low	high	low	clear
e	low	high	low	unclear

Fifty-two years old, leiomyoma, low T2WI intensity, high T1WI intensity, low DWI intensity, clear margin, LDH 189IU/L.

Supplementary Figure S29. Case29 A) T2WI, B) T1WI, C) DWI, D) image evaluations of Readers a–e.

A

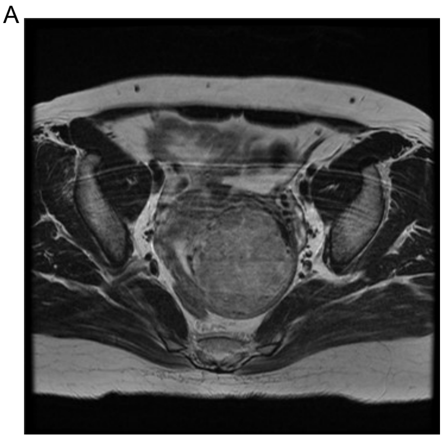

B

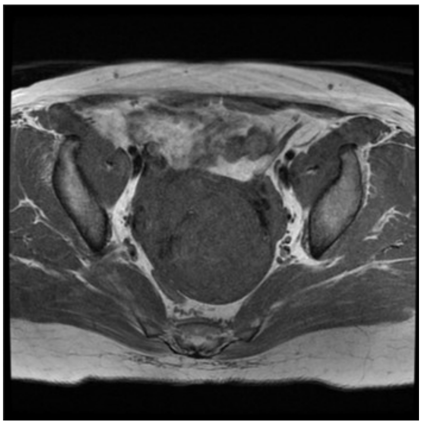

C

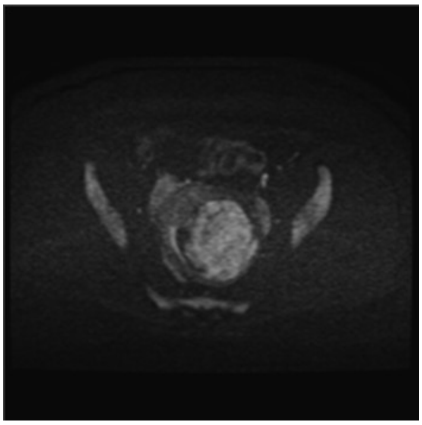

D

	T2	T1	DWI	margin
a	high	low	high	clear
b	high	high	low	clear
c	high	low	high	clear
d	high	low	high	clear
e	high	low	low	unclear

Forty-four years old, leiomyoma, high T2WI intensity, low T1WI intensity, high DWI intensity, clear margin, LDH 162IU/L. We reviewed the images and determined that the T2WI was Oguchi 4.

Supplementary Figure S30. Case30 A) T2WI, B) T1WI, C) DWI, D) image evaluations of Readers a–e.

A

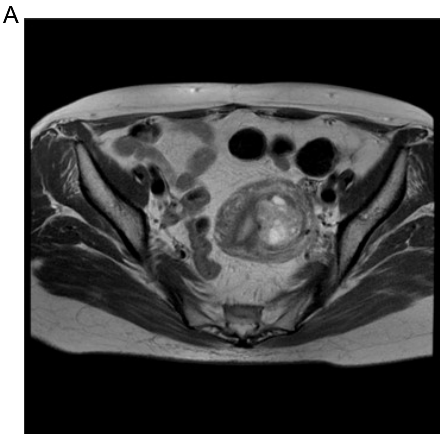

B

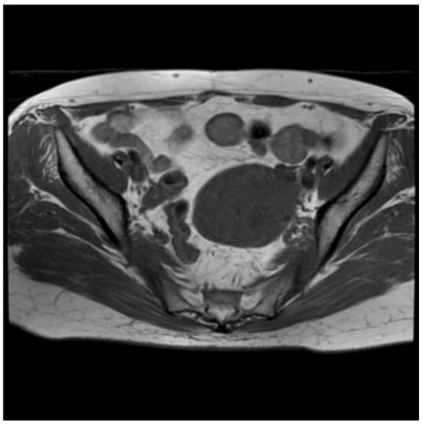

C

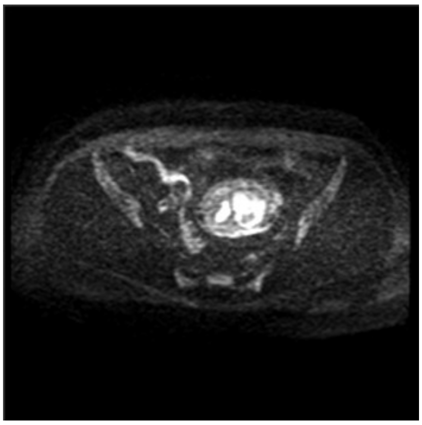

D

	T2	T1	DWI	margin
a	high	low	high	clear
b	high	low	low	clear
c	high	low	high	unclear
d	high	low	high	unclear
e	high	low	low	unclear

Forty years old, leiomyoma, high T2WI intensity, low T1WI intensity, high DWI intensity, unclear margin, LDH 186IU/L.

Supplementary Figure S31. Case31 A) T2WI, B) T1WI, C) DWI, D) image evaluations of Readers a–e.

A

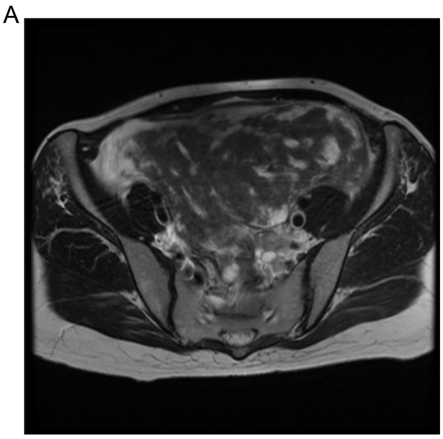

B

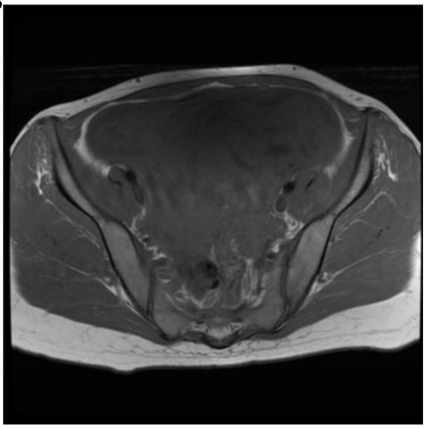

C

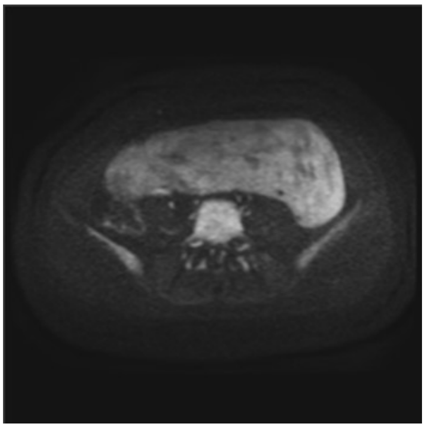

D

	T2	T1	DWI	margin
a	high	low	high	clear
b	low	low	high	clear
c	high	low	high	unclear
d	high	low	high	unclear
e	low	low	high	clear

Forty-one years old, leiomyoma, low T2WI intensity, low T1WI intensity, high DWI intensity, clear margin, LDH 179IU/L. We reviewed the images and determined that the T2WI was Oguchi 3.

Supplementary Figure S32. Case32 A) T2WI, B) T1WI, C) DWI, D) image evaluations of Readers a–e.

A

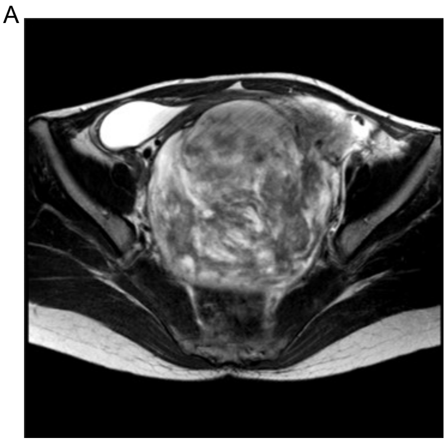

B

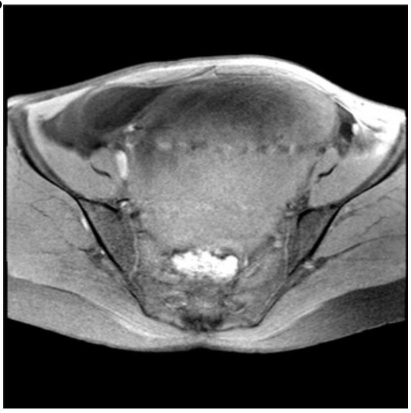

C

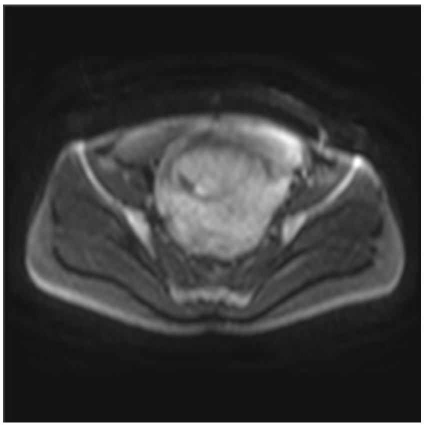

D

	T2	T1	DWI	margin
a	high	low	high	clear
b	low	low	low	clear
c	high	low	high	unclear
d	high	low	high	unclear
e	low	low	high	clear

Thirty-eight years old, leiomyoma, low T2WI intensity, low T1WI intensity, high DWI intensity, clear margin, LDH 187IU/L. We reviewed the images and determined that the T2WI was Oguchi 3.

Supplementary Figure S33. Case33 A) T2WI, B) T1WI, C) DWI, D) image evaluations of Readers a–e.

A

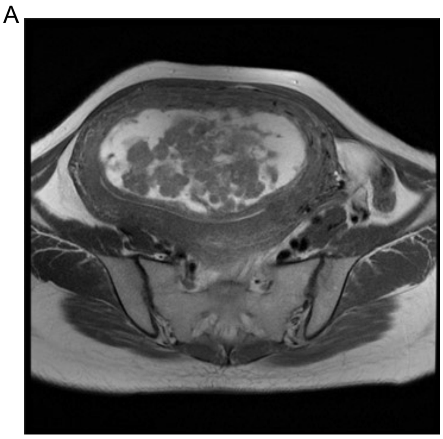

B

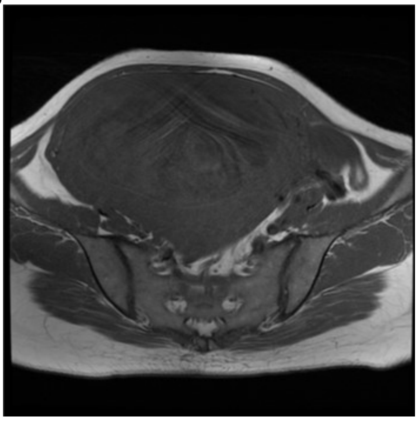

C

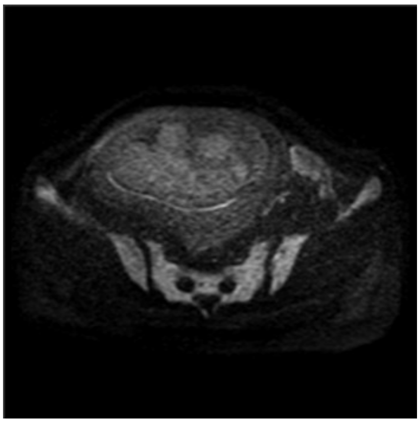

D

	T2	T1	DWI	margin
a	high	low	high	clear
b	low	high	high	clear
c	high	high	high	unclear
d	high	low	high	clear
e	low	low	low	clear

Forty-eight years old, leiomyoma, low T2WI intensity, low T1WI intensity, high DWI intensity, clear margin, LDH 180IU/L. We reviewed the images and determined that the T2WI was Oguchi 3.

Supplementary Figure S34. Case34 A) T2WI, B) T1WI, C) DWI, D) image evaluations of Readers a–e.

A

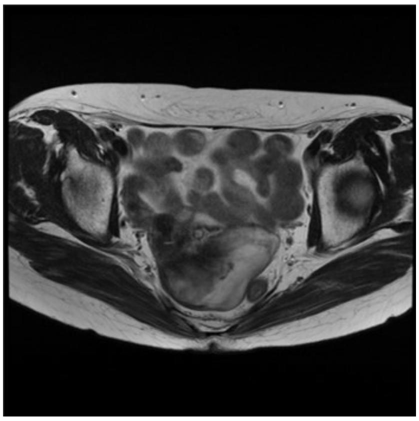

B

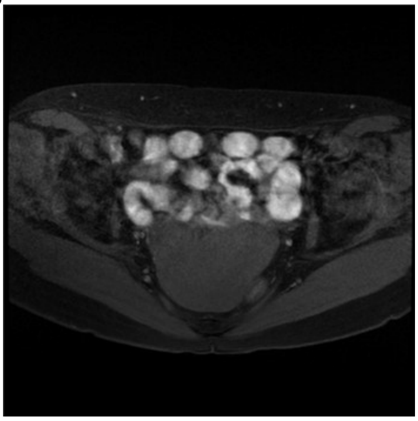

C

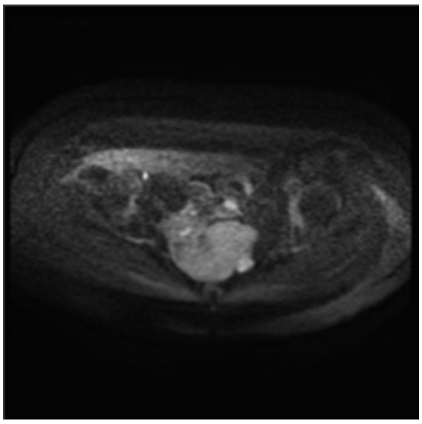

D

	T2	T1	DWI	margin
a	high	low	high	clear
b	high	low	high	clear
c	high	low	low	unclear
d	high	low	high	unclear
e	high	low	low	unclear

Seventy years old, leiomyoma, high T2WI intensity, low T1WI intensity, high DWI intensity, clear margin, LDH 192IU/L.

Supplementary Figure S35. Case35 A) T2WI, B) T1WI, C) DWI, D) image evaluations of Readers a–e.

A

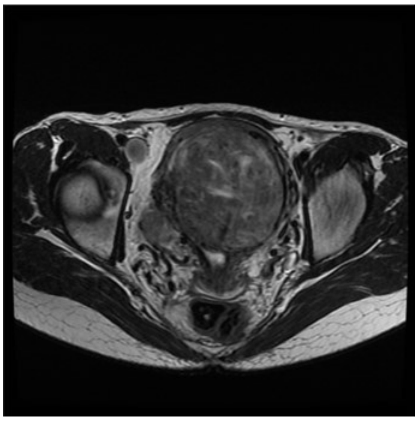

B

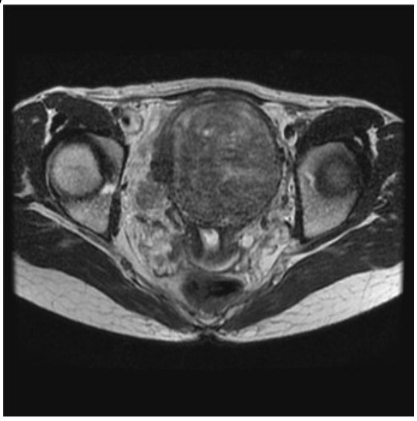

C

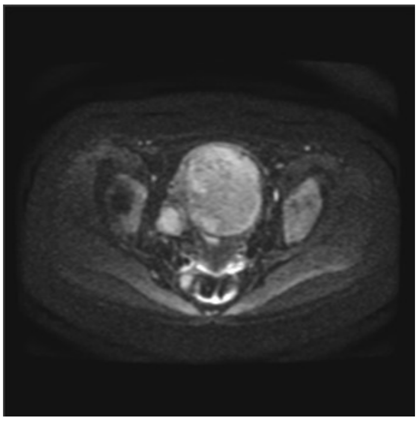

D

	T2	T1	DWI	margin
a	high	low	high	clear
b	low	low	high	clear
c	high	low	high	clear
d	high	high	high	clear
e	low	high	high	clear

Thirty-four years old, leiomyoma, low T2WI intensity, low T1WI intensity, high DWI intensity, clear margin, LDH 182IU/L. We reviewed the images and determined that the T2WI was Oguchi 3.

Supplementary Figure S36. Case36 A) T2WI, B) T1WI, C) DWI, D) image evaluations of Readers a–e.

A

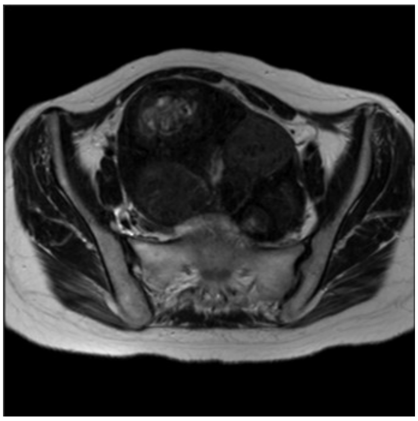

B

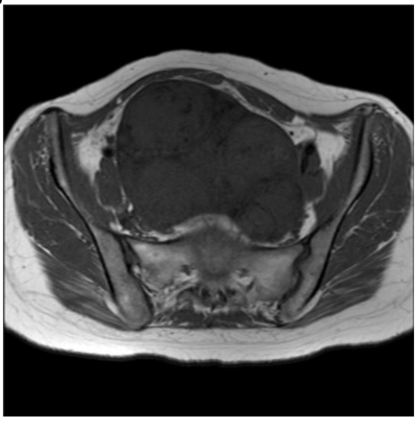

C

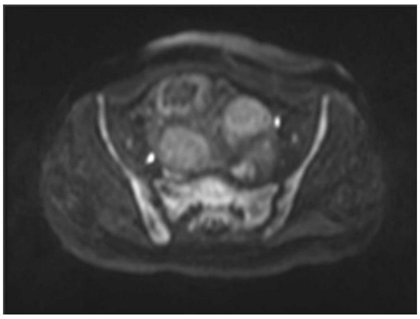

D

	T2	T1	DWI	margin
a	high	low	high	clear
b	low	low	low	clear
c	low	low	high	clear
d	high	low	high	unclear
e	low	low	low	clear

Forty years old, leiomyoma, low T2WI intensity, low T1WI intensity, high DWI intensity, clear margin, LDH 185IU/L. We reviewed the images and determined that the T2WI was Oguchi 3.

Supplementary Figure S37. Case37 A) T2WI, B) T1WI, C) DWI, D) image evaluations of Readers a–e.

A

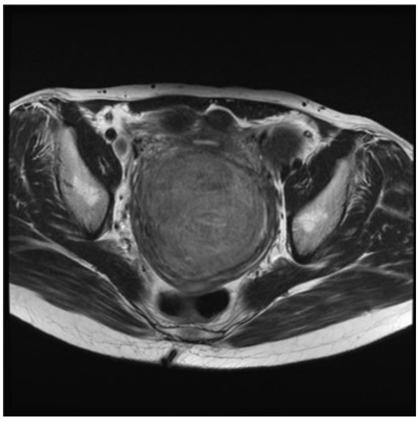

B

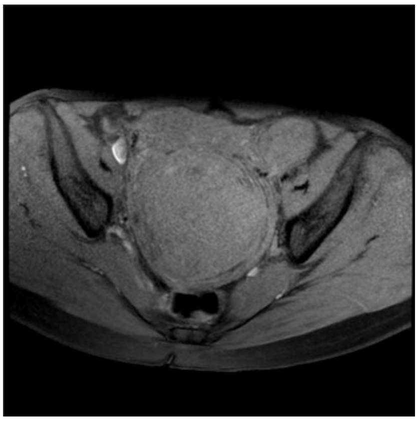

C

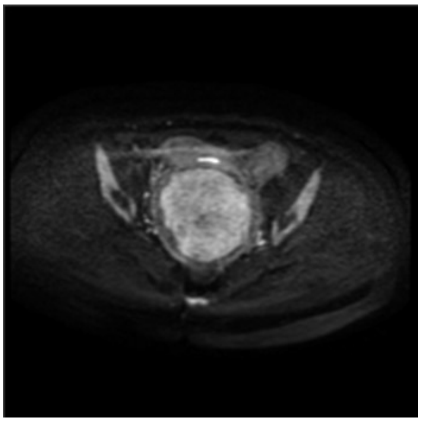

D

	T2	T1	DWI	margin
a	high	low	high	clear
b	high	low	high	clear
c	high	low	high	clear
d	high	low	high	clear
e	high	high	high	clear

Thirty-seven years old, leiomyoma, high T2WI intensity, low T1WI intensity, high DWI intensity, clear margin, LDH 165IU/L.

Supplementary Figure S38. Case38 A) T2WI, B) T1WI, C) DWI, D) image evaluations of Readers a–e.

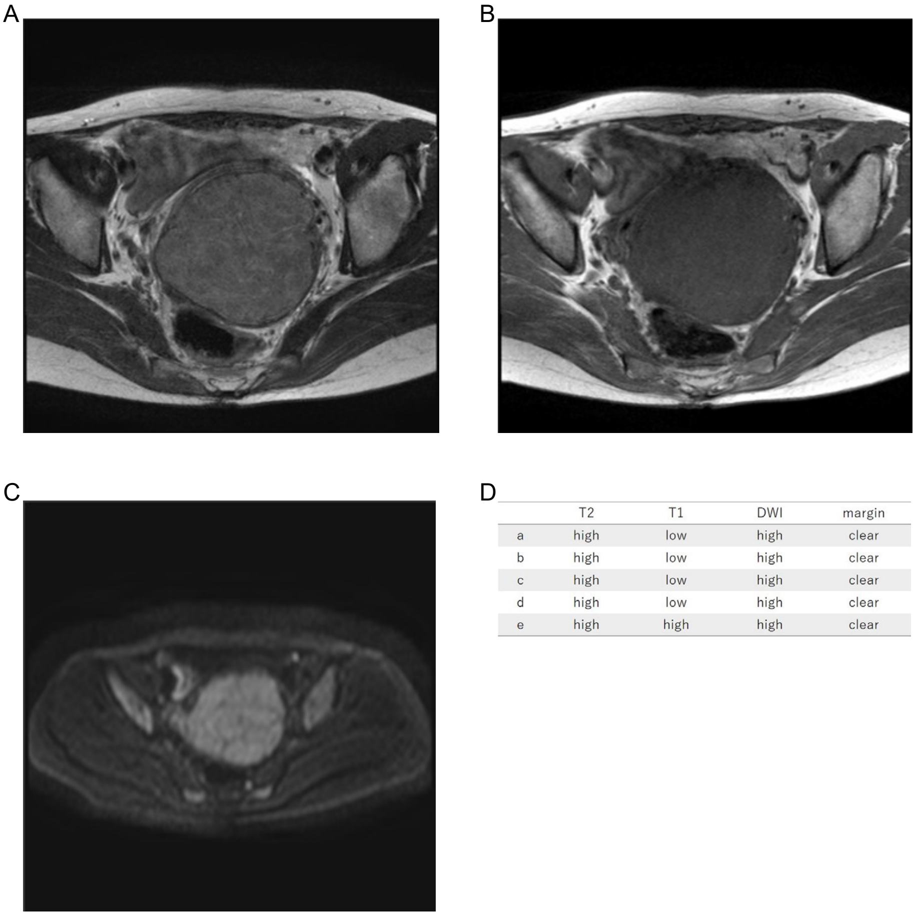

D

	T2	T1	DWI	margin
a	high	low	high	clear
b	high	low	high	clear
c	high	low	high	clear
d	high	low	high	clear
e	high	high	high	clear

Thirty-four years old, leiomyoma, high T2WI intensity, low T1WI intensity, high DWI intensity, clear margin, LDH 142IU/L.

Supplementary Figure S39. Case39 A) T2WI, B) T1WI, C) DWI, D) image evaluations of Readers a–e.

A

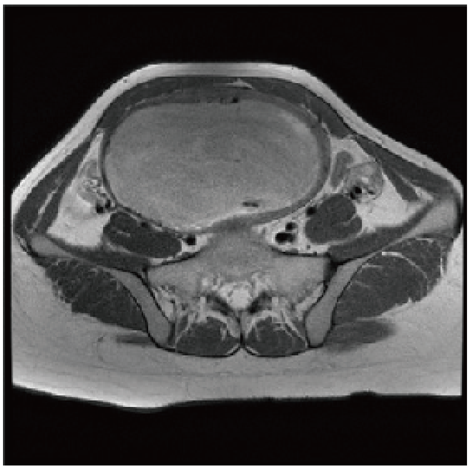

B

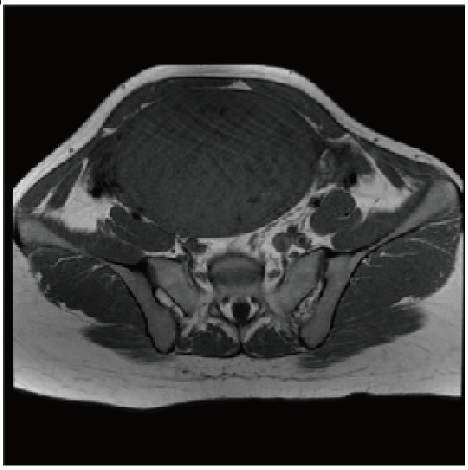

C

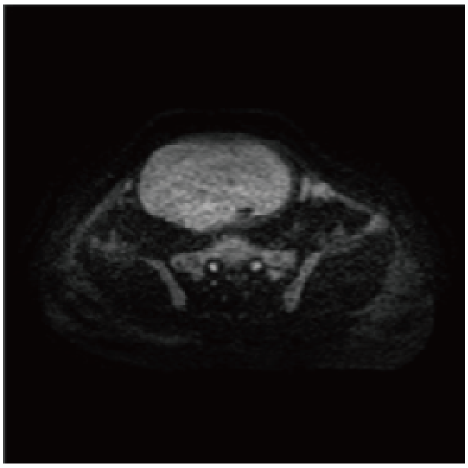

D

	T2	T1	DWI	margin
a	high	low	high	clear
b	high	low	high	clear
c	high	low	high	clear
d	high	low	high	clear
e	high	low	high	clear

Thirty-two years old, leiomyoma, high T2WI intensity, low T1WI intensity, high DWI intensity, clear margin, LDH 159IU/L.

Supplementary Figure S40. Case40 A) T2WI, B) T1WI, C) DWI, D) image evaluations of Readers a–e.

A

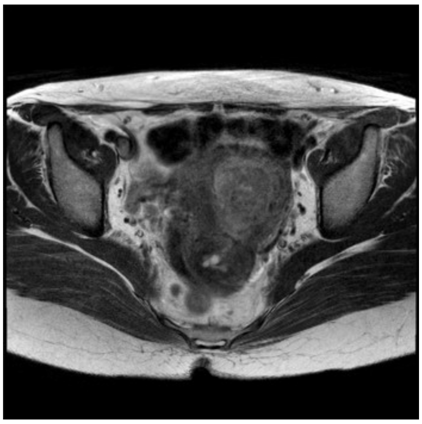

B

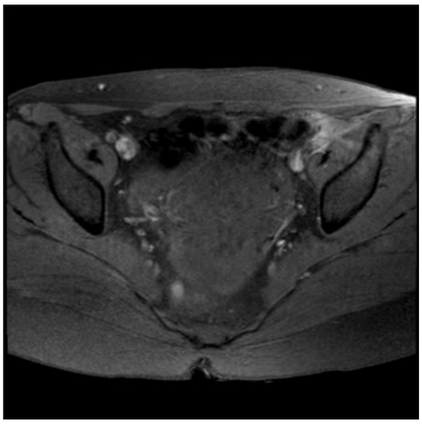

C

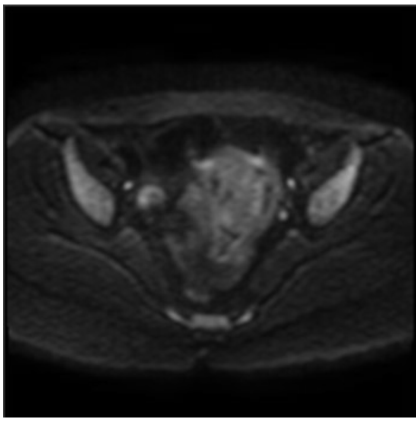

D

	T2	T1	DWI	margin
a	high	low	high	clear
b	low	low	low	clear
c	high	low	low	clear
d	high	low	low	clear
e	high	low	low	unclear

Forty-four years old, leiomyoma, low T2WI intensity, low T1WI intensity, low DWI intensity, clear margin, LDH 167IU/L. We reviewed the images and determined that the T2WI was Oguchi 3.

Supplementary Figure S41. Case41 A) T2WI, B) T1WI, C) DWI, D) image evaluations of Readers a–e.

A

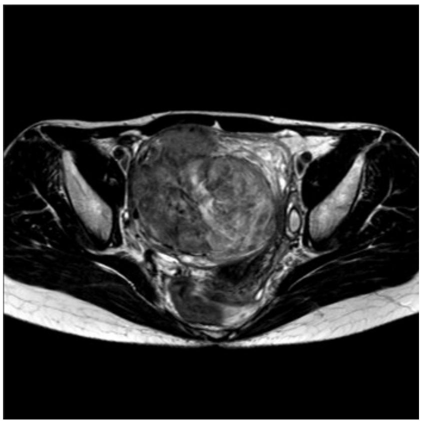

B

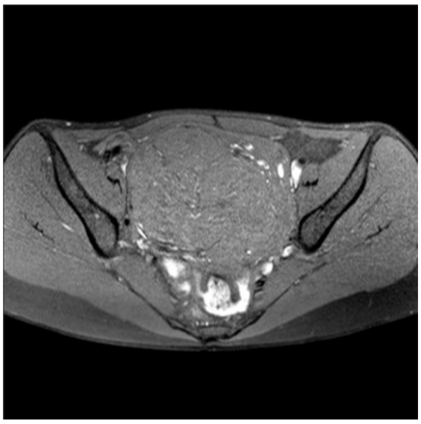

C

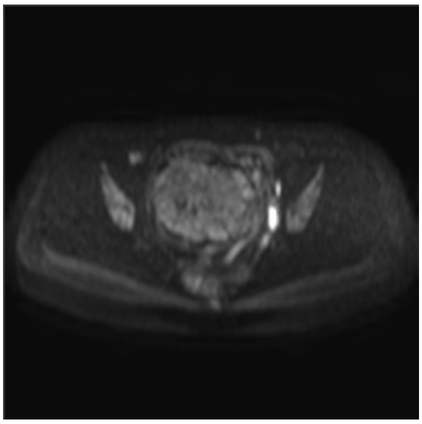

D

	T2	T1	DWI	margin
a	high	low	low	clear
b	low	low	high	clear
c	high	low	high	clear
d	low	low	low	clear
e	low	low	high	clear

Thirty-two years old, leiomyoma, low T2WI intensity, low T1WI intensity, high DWI intensity, clear margin, LDH 170IU/L. We reviewed the images and determined that the T2WI was Oguchi 3.

Supplementary Figure S42. Case42 A) T2WI, B) T1WI, C) DWI, D) image evaluations of Readers a–e.

A

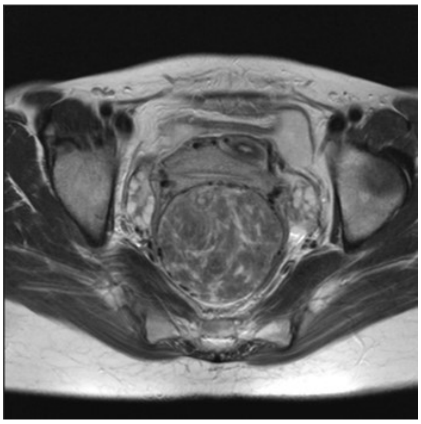

B

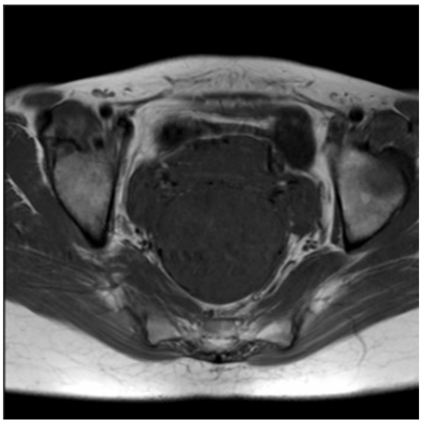

C

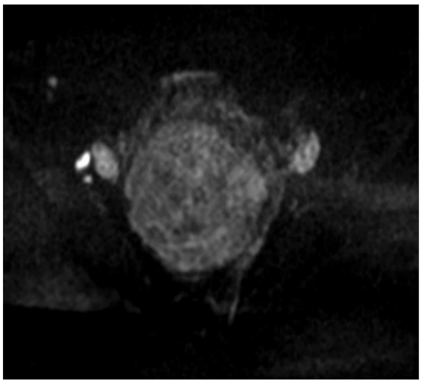

D

	T2	T1	DWI	margin
a	high	low	low	clear
b	low	low	high	clear
c	high	low	low	clear
d	high	low	high	clear
e	low	low	low	clear

Thirty years old, leiomyoma, low T2WI intensity, low T1WI intensity, low DWI intensity, clear margin, LDH 139IU/L. We reviewed the images and determined that the T2WI was Oguchi 3.

Supplementary Figure S43. Case43 A) T2WI, B) T1WI, C) DWI, D) image evaluations of Readers a–e.

A

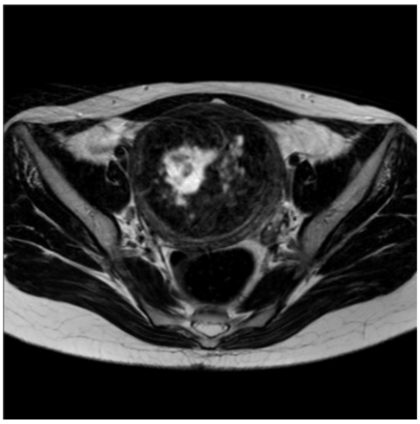

B

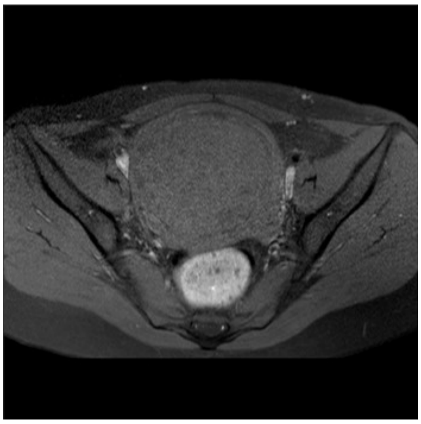

C

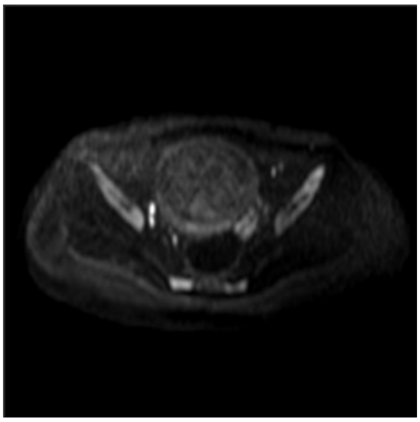

D

	T2	T1	DWI	margin
a	high	low	low	clear
b	low	low	low	clear
c	high	low	low	clear
d	high	low	low	clear
e	low	low	low	clear

Thirty-three years old, leiomyoma, low T2WI intensity, low T1WI intensity, low DWI intensity, clear margin, LDH 163IU/L. We reviewed the images and determined that the T2WI was Oguchi 3.

Supplementary Figure S44. Case44 A) T2WI, B) T1WI, C) DWI, D) image evaluations of Readers a–e.

A

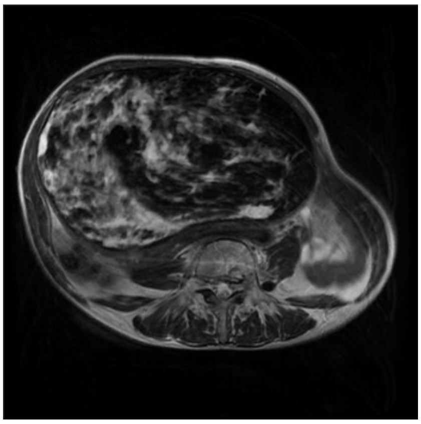

B

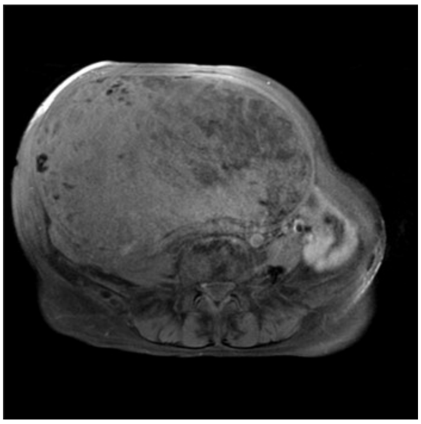

C

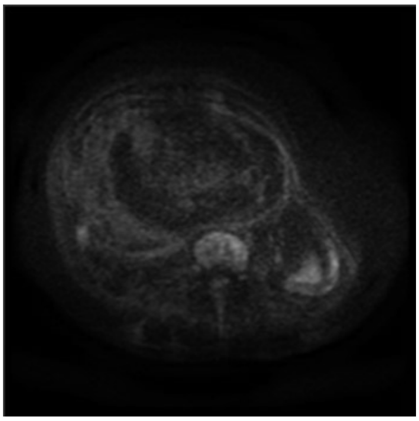

D

	T2	T1	DWI	margin
a	high	low	low	clear
b	low	low	low	clear
c	high	low	low	clear
d	high	low	low	clear
e	low	low	low	clear

Sixty-eight years old, leiomyoma, low T2WI intensity, low T1WI intensity, low DWI intensity, clear margin, LDH 222IU/L. We reviewed the images and determined that the T2WI was Oguchi 3.

Supplementary Figure S45. Case45 A) T2WI, B) T1WI, C) DWI, D) image evaluations of Readers a–e.

A

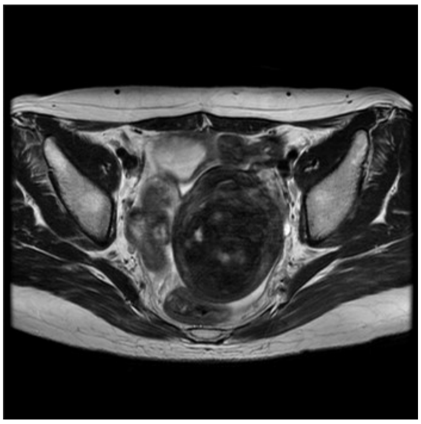

B

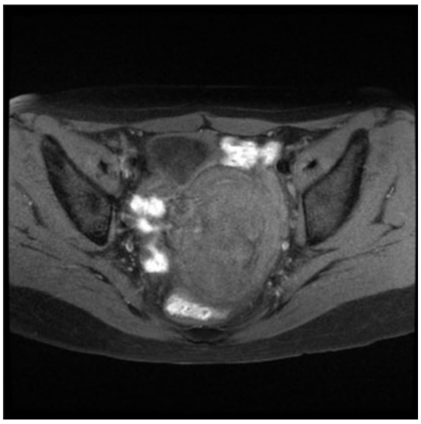

C

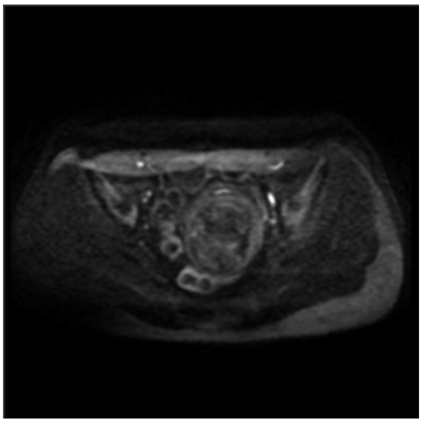

D

	T2	T1	DWI	margin
a	high	low	low	clear
b	low	low	low	clear
c	low	low	low	clear
d	low	low	low	clear
e	low	low	low	clear

Forty-two years old leiomyoma, low T2WI intensity, low T1WI intensity, low DWI intensity, clear margin, LDH 148IU/L. We reviewed the images and determined that the T2WI was Oguchi 3.

Supplementary Figure S46. Case46 A) T2WI, B) T1WI, C) DWI, D) image evaluations of Readers a–e.

A

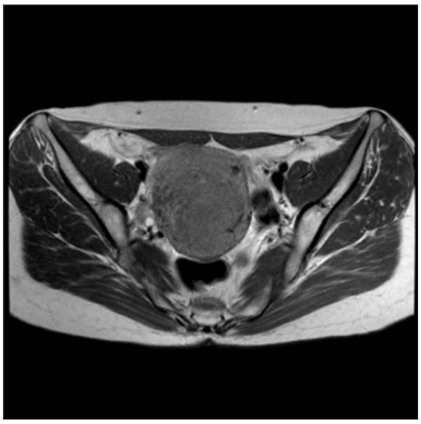

B

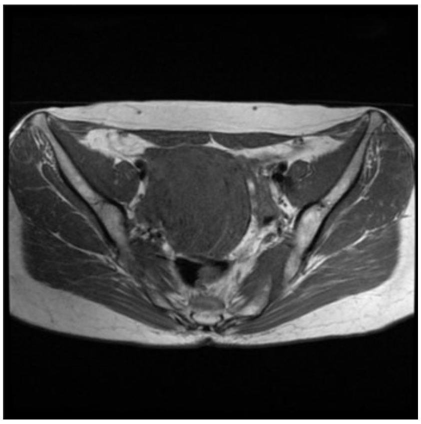

C

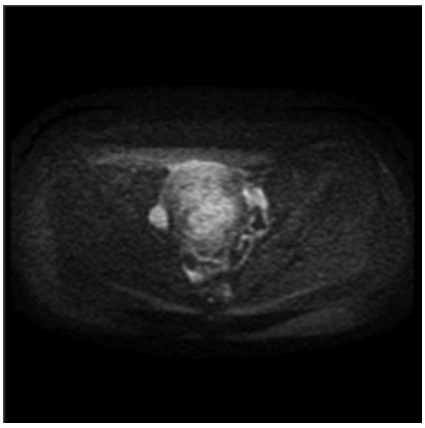

D

	T2	T1	DWI	margin
a	low	low	high	clear
b	high	low	high	clear
c	high	low	low	clear
d	low	low	high	clear
e	low	low	high	clear

Forty-three years old. leiomyoma, low T2WI intensity, low T1WI intensity, high DWI intensity, clear margin, LDH 191IU/L.

Supplementary Figure S47. Case47 A) T2WI, B) T1WI, C) DWI, D) image evaluations of Readers a–e.

A

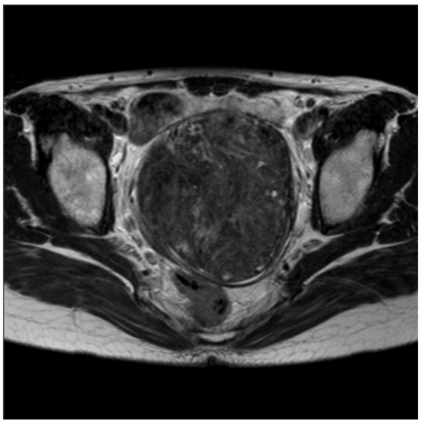

B

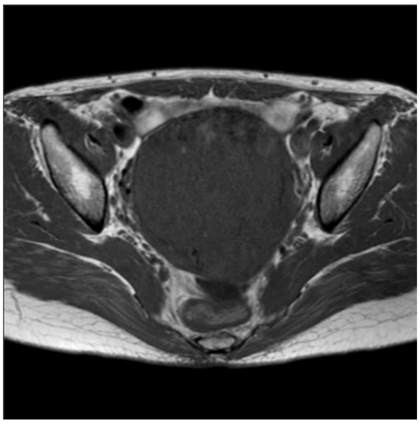

C

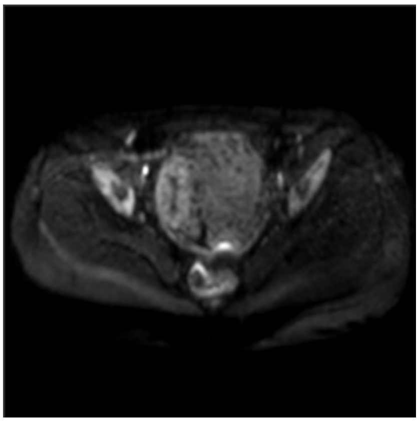

D

	T2	T1	DWI	margin
a	low	low	high	clear
b	low	high	high	clear
c	low	low	low	clear
d	low	low	high	clear
e	low	low	low	clear

Forty-eight years old, leiomyoma, low T2WI intensity, low T1WI intensity, high DWI intensity, clear margin, LDH 185IU/L.

Supplementary Figure S48. Case48 A) T2WI, B) T1WI, C) DWI, D) image evaluations of Readers a–e.

A

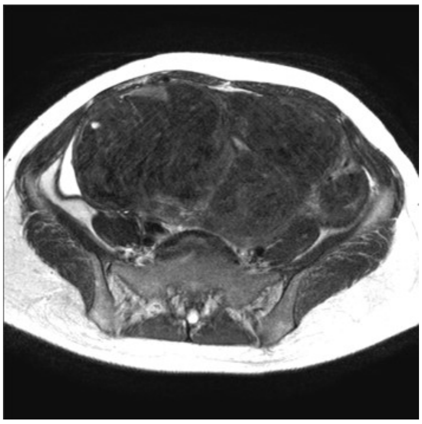

B

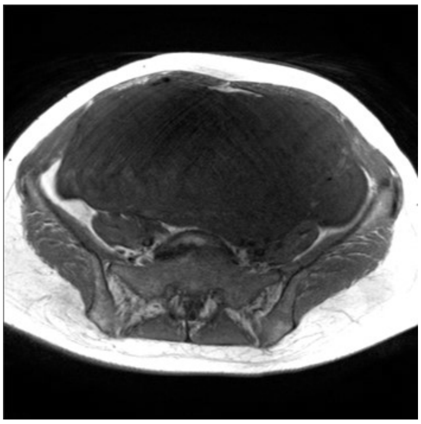

C

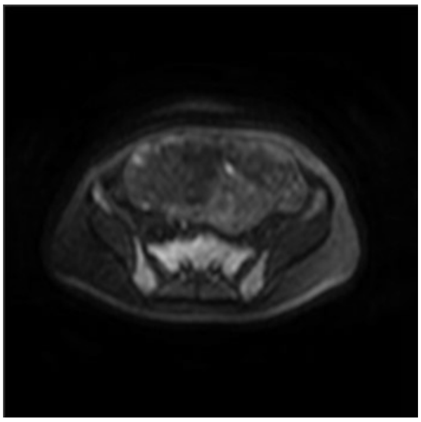

D

	T2	T1	DWI	margin
a	low	low	high	clear
b	low	low	low	clear
c	low	low	low	clear
d	low	high	high	clear
e	low	low	low	clear

Forty-three years old, leiomyoma, low T2WI intensity, low T1WI intensity, low DWI intensity, clear margin, LDH 217IU/L.

Supplementary Figure S49. Case49 A) T2WI, B) T1WI, C) DWI, D) image evaluations of Readers a–e.

A

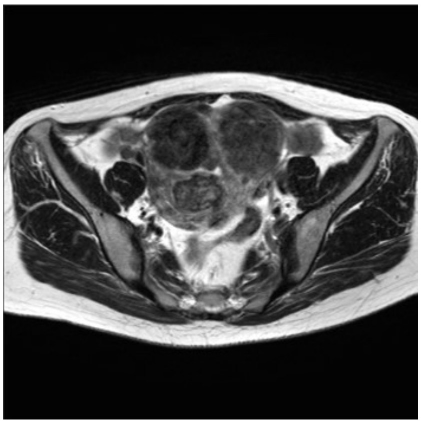

B

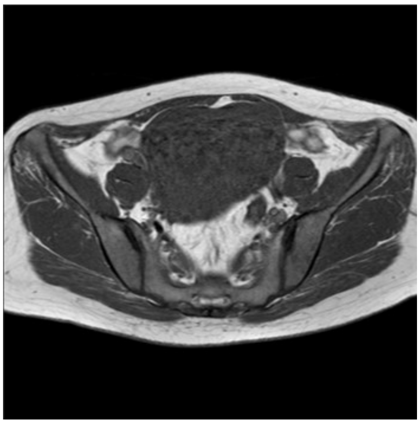

C

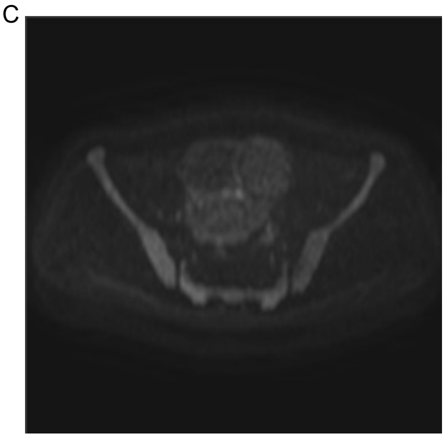

D

	T2	T1	DWI	margin
a	low	low	low	clear
b	high	low	low	clear
c	low	low	low	clear
d	low	low	low	clear
e	low	low	low	clear

Forty-seven years old, leiomyoma, low T2WI intensity, low T1WI intensity, low DWI intensity, clear margin, LDH 179IU/L.

Supplementary Figure S50. Case50 A) T2WI, B) T1WI, C) DWI, D) image evaluations of Readers a–e.

A

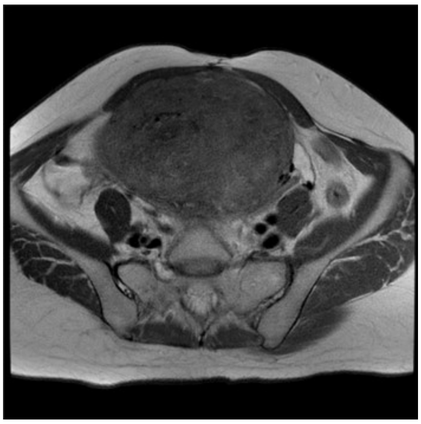

B

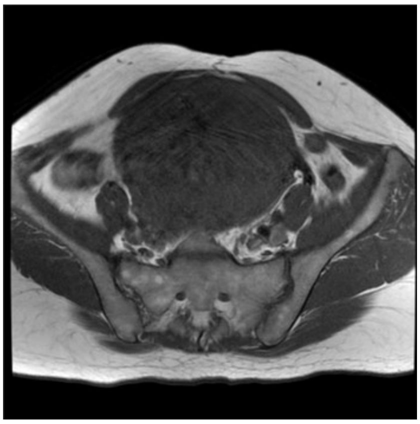

C

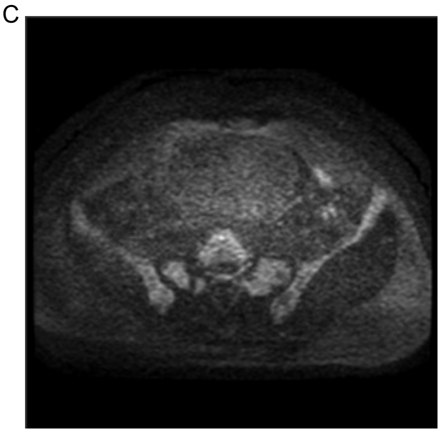

D

	T2	T1	DWI	margin
a	low	low	low	clear
b	low	low	high	clear
c	low	low	low	clear
d	low	low	low	clear
e	low	low	low	clear

Forty-eight years old, leiomyoma, low T2WI intensity, low T1WI intensity, low DWI intensity, clear margin, LDH 184IU/L.

Supplementary Figure S51. Case5 A) T2WI, B) T1WI, C) DWI, D) image evaluations of Readers a–e.

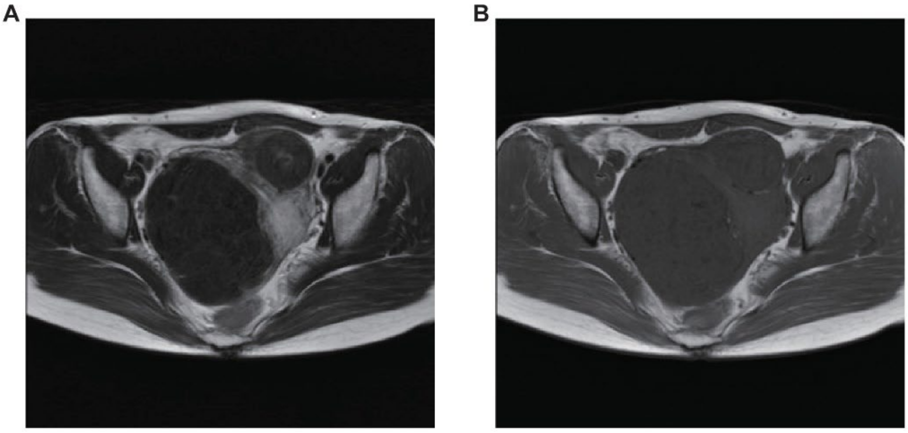

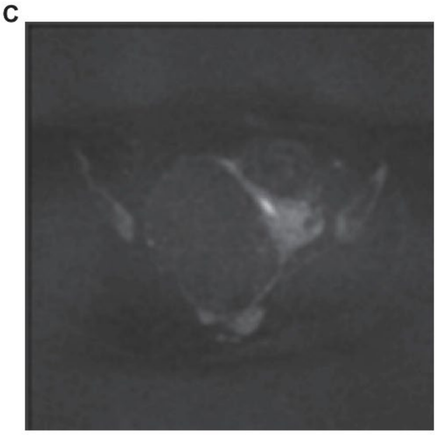

D

	T2	T1	DWI	margin
a	low	low	low	clear
b	low	low	low	clear
c	low	low	low	clear
d	low	low	low	clear
e	low	low	high	clear

Forty-one years old, leiomyoma, low T2WI intensity, low T1WI intensity, low DWI intensity, clear margin, LDH 223IU/L.

Supplementary Figure S52. Case52 A) T2WI, B) T1WI, C) DWI, D) image evaluations of Readers a–e.

A

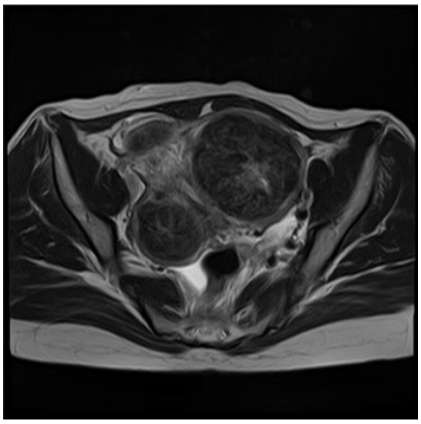

B

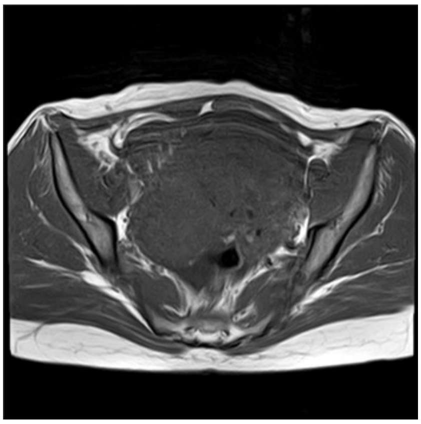

C

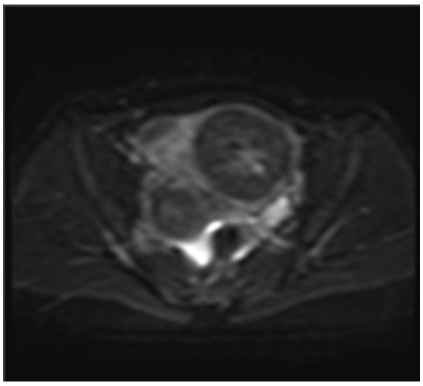

D

	T2	T1	DWI	margin
a	low	low	low	clear
b	low	low	low	clear
c	low	low	low	clear
d	low	low	low	clear
e	low	low	low	clear

Forty-nine years old, leiomyoma, low T2WI intensity, low T1WI intensity, low DWI intensity, clear margin, LDH 235IU/L.

Supplementary Figure S53. Case53 A) T2WI, B) T1WI, C) DWI, D) image evaluations of Readers a–e.

A

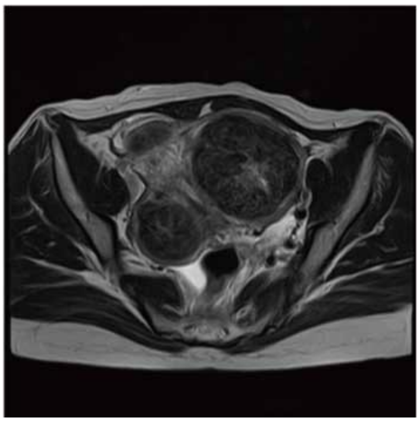

B

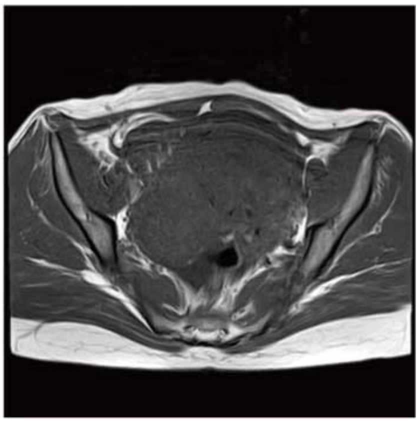

C

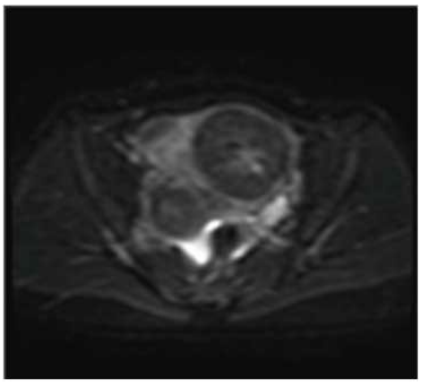

D

	T2	T1	DWI	margin
a	low	low	low	clear
b	low	low	low	clear
c	low	low	low	clear
d	low	low	low	clear
e	low	low	low	clear

Thirty-nine years old, leiomyoma, low T2WI intensity, low T1WI intensity, low DWI intensity, clear margin, LDH 179IU/L.

Supplementary Figure S54. Case54 A) T2WI, B) T1WI, C) DWI, D) image evaluations of Readers a–e.

A

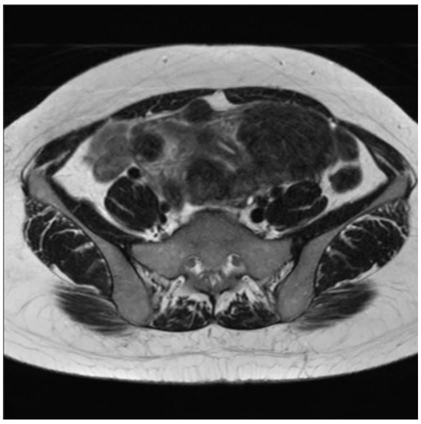

B

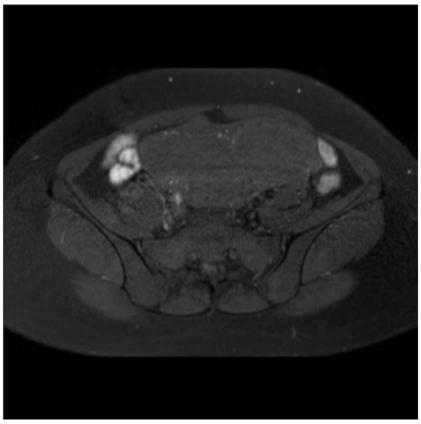

C

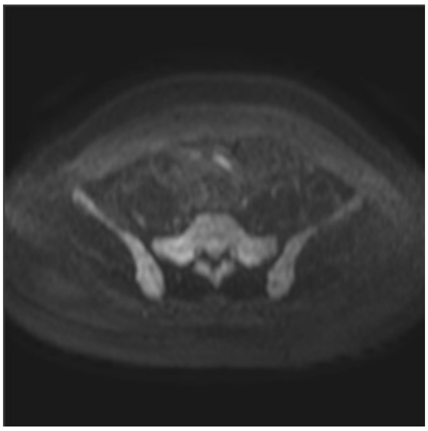

D

	T2	T1	DWI	margin
a	low	low	low	clear
b	low	low	low	clear
c	low	low	low	clear
d	low	low	low	clear
e	low	low	low	unclear

Thirty-eight years old, leiomyoma, low T2WI intensity, low T1WI intensity, low DWI intensity, clear margin, LDH 205IU/L.

Supplementary Figure S55. Case55 A) T2WI, B) T1WI, C) DWI, D) image evaluations of Readers a–e.

A

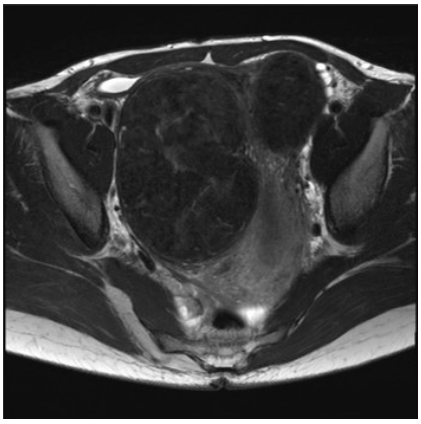

B

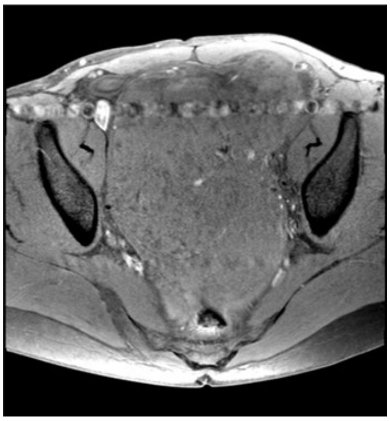

C

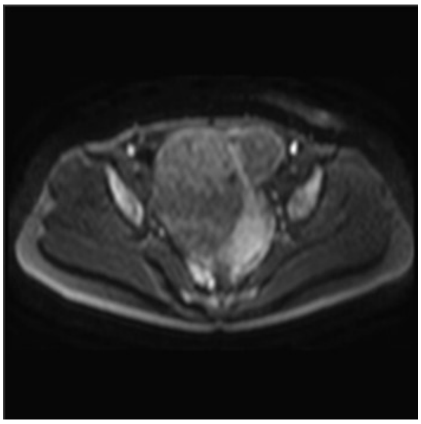

D

	T2	T1	DWI	margin
a	low	low	low	clear
b	low	low	low	clear
c	low	low	low	clear
d	low	low	low	clear
e	low	low	low	clear

Thirty-six years old, leiomyoma, low T2WI intensity, low T1WI intensity, low DWI intensity, clear margin, LDH 162IU/L.

Supplementary Figure S56. Case56 A) T2WI, B) T1WI, C) DWI, D) image evaluations of Readers a–e.

A

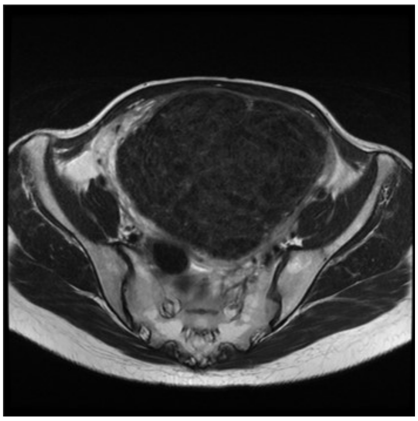

B

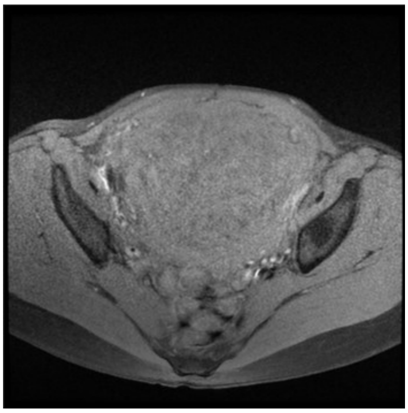

C

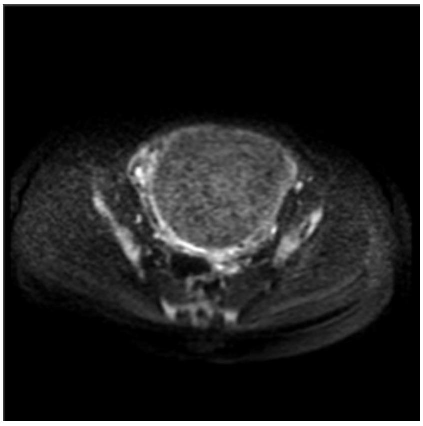

D

	T2	T1	DWI	margin
a	low	low	low	clear
b	low	low	low	clear
c	low	low	low	clear
d	low	low	low	clear
e	low	low	low	clear

Thirty-six years old, leiomyoma, low T2WI intensity, low T1WI intensity, low DWI intensity, clear margin, LDH 174IU/L.

Supplementary Figure S57. Case57 A) T2WI, B) T1WI, C) DWI, D) image evaluations of Readers a–e.

A

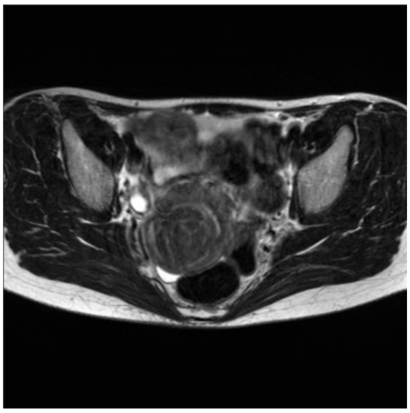

B

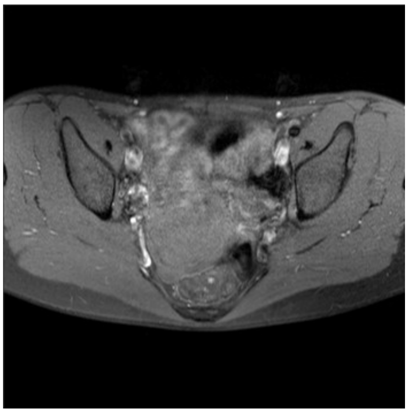

C

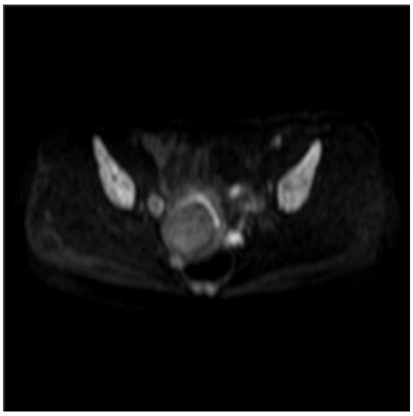

D

	T2	T1	DWI	margin
a	low	low	low	clear
b	low	low	low	clear
c	low	low	low	clear
d	low	low	low	clear
e	low	low	low	clear

Forty-two years old, low T2WI intensity, low T1WI intensity, low DWI intensity, clear margin, LDH 176IU/L.

Supplementary Figure S58. Case58 A) T2WI, B) T1WI, C) DWI, D) image evaluations of Readers a–e.

A

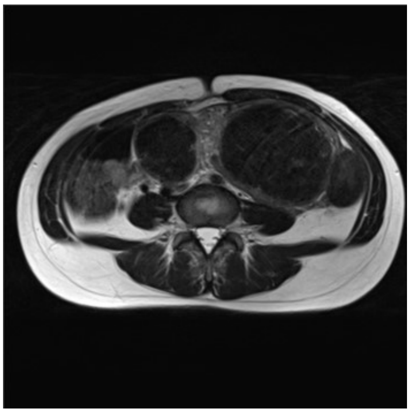

B

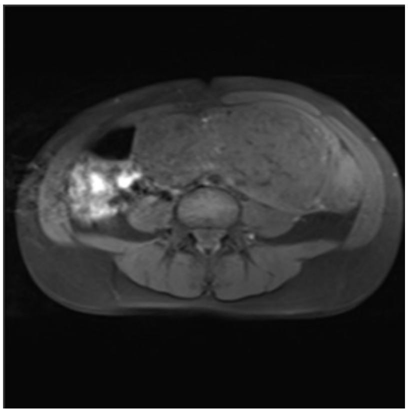

C

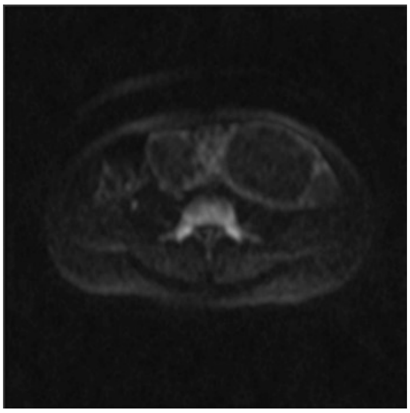

D

	T2	T1	DWI	margin
a	low	low	low	clear
b	low	low	low	clear
c	low	low	low	clear
d	low	low	low	clear
e	low	low	low	clear

Twenty-seven years old, leiomyoma, low T2WI intensity, low T1WI intensity, low DWI intensity, clear margin, LDH 181IU/L.

Supplementary Figure S59. Case59 A) T2WI, B) T1WI, C) DWI, D) image evaluations of Readers a–e.

A B

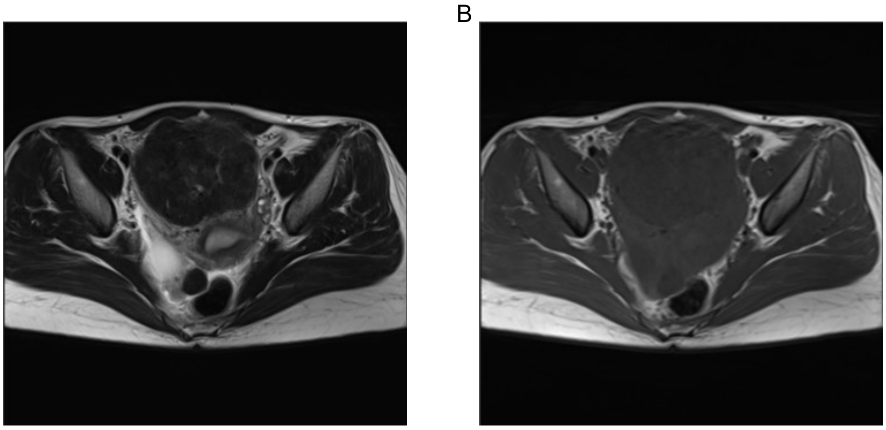

C

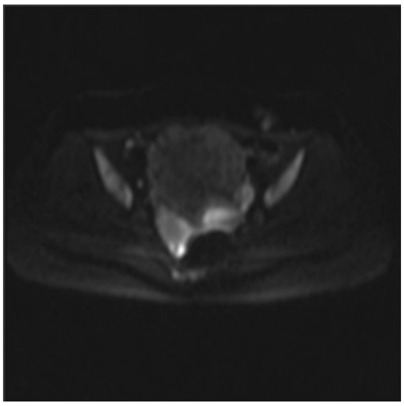

D

	T2	T1	DWI	margin
a	low	low	low	clear
b	low	low	low	clear
c	low	low	low	clear
d	low	low	low	clear
e	low	low	low	clear

Thirty-five years old, leiomyoma, low T2WI intensity, low T1WI intensity, low DWI intensity, clear margin, LDH 138IU/L.

Supplementary Figure S60. Case60 A) T2WI, B) T1WI, C) DWI, D) image evaluations of Readers a–e.

A

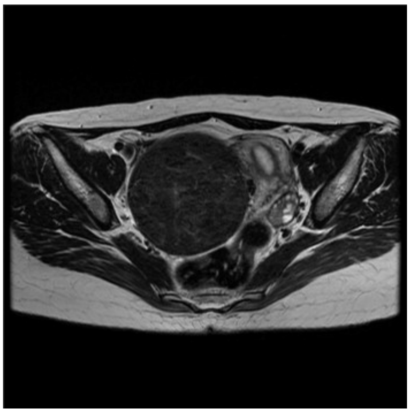

B

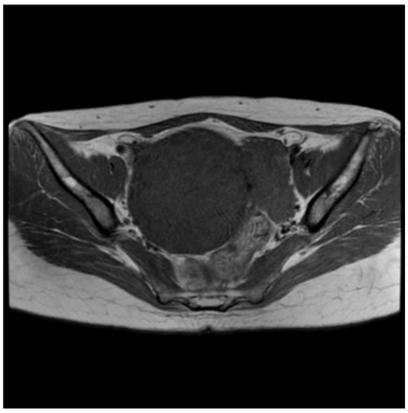

C

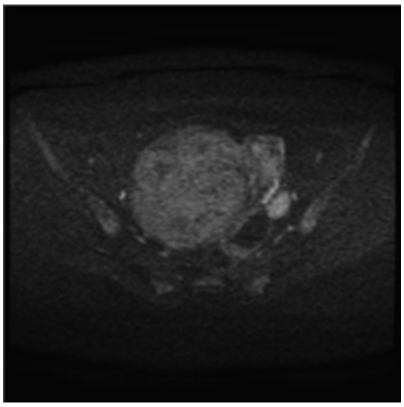

D

	T2	T1	DWI	margin
a	low	low	low	clear
b	low	low	low	clear
c	low	low	low	clear
d	low	low	low	clear
e	low	low	low	clear

Twenty-six years old, leiomyoma, low T2WI intensity, low T1WI intensity, low DWI intensity, clear margin, LDH 183IU/L.

Supplementary Figure S61. Case61 A) T2WI, B) T1WI, C) DWI, D) image evaluations of Readers a–e.

A

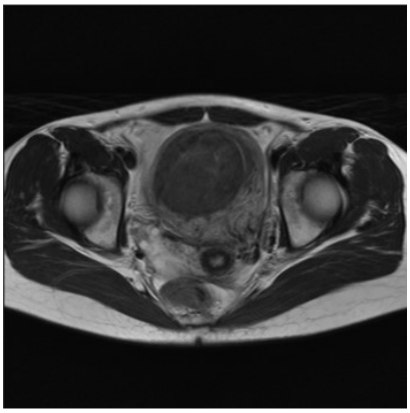

B

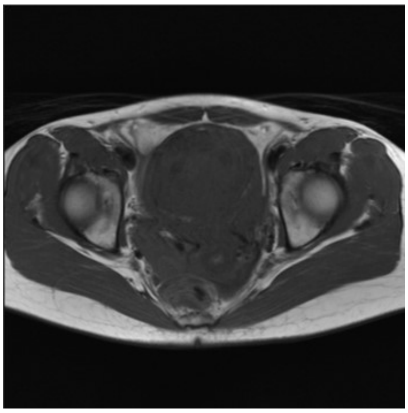

C

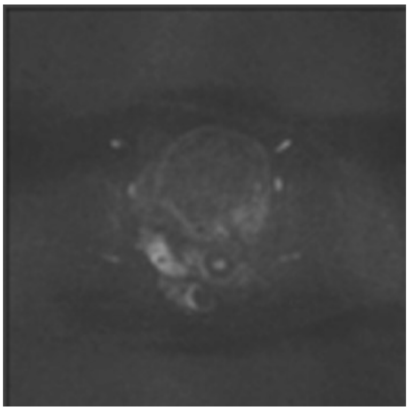

D

	T2	T1	DWI	margin
a	low	low	low	clear
b	low	low	low	clear
c	low	low	low	clear
d	low	low	low	clear
e	low	low	low	clear

Twenty-five years old, leiomyoma, low T2WI intensity, low T1WI intensity, low DWI intensity, clear margin, LDH 178IU/L.
